# Differences in the burden and temporal trend of stroke in the Belt and Road Initiative countries, 1990-2021

**DOI:** 10.1515/med-2026-1532

**Published:** 2026-09-24

**Authors:** Zheng Luo, Yue Zhang, Huihui Lv, Jinyi Wu, Xiaopan Li

**Affiliations:** Department of Neurology, Shanghai University of Medicine & Health Sciences Affiliated Zhoupu Hospital, Shanghai, China; Department of Epidemiology, School of Public Health, Shanxi Medical University, Taiyuan, China; Department of Neurology, Yueyang Hospital of Integrated Traditional Chinese and Western Medicine, Shanghai, China; Department of Public Health, Wuhan Fourth Hospital Wuhan, China; Department of Health Management Center, Zhongshan Hospital, Shanghai Medical College of Fudan University, Shanghai, China; Shanghai Key Laboratory of Meteorology and Health, Shanghai, China

**Keywords:** “B&R” countries, stroke, burden of disease, years lived with disability (YLDs), disability-adjusted life years

## Abstract

**Objectives:**

To analyze the differences in the burden and trend of disability caused by stroke in “Belt and Road” (“B&R”) countries from 1990 to 2021.

**Methods:**

We applied GBD 2021 to compare stroke incidence, mortality, the years lived with disability (YLDs) and disability-adjusted life-years (DALYs) from 1990 to 2021 in the “B&R” countries. The average annual percent change (AAPC) was used to analyze the temporal trends of age-standardized YLDs rate of stroke from 1990 to 2021 by Joinpoint regression analysis.

**Results:**

China, India and Indonesia had the highest absolute stroke numbers of stroke. From 1990 to 2021, the AAPC values of age-standardized metrics showed a downward trend in most of “B&R” countries. Turkmenistan had the highest increase in AAPCs of age-standardized YLDs rate for men (AAPC=0.60 %), while for women Philippines had the highest increase (AAPC=0.57 %). For under 15 years, YLDs due to stroke in most member states showed a downward trend, while increasing trends were noted in Saudi Arabia and Turkmenistan. Globally, metabolic, environmental/occupational and behavioral risk factors showed significant reductions (AAPC: −0.12 %, −0.92 %, and −0.53 %, respectively).

**Conclusions:**

The overall burden of stroke remains enormous, with regional, age and gender disparities. Enhanced health cooperation among B&R countries is warranted.

## Introduction

First proposed by Chinese President Xi Jinping’s in 2013, the Chinese government initiates the Belt and Road Initiative (BRI) to promote trade, infrastructure development and commercial partnerships among 66 countries in Asia, Africa, South America and Europe [1], 2]. Despite the BRI’s focus on economic development and infrastructure investment, its impact on global health is emerging [3]. In 2017, the Chinese government proposed the “Health Silk Road” (HSR) initiative to strengthen global health cooperation. Under the framework of HSR, China and partner countries have prioritized strengthening health systems through training health professionals, setting up disease control centers, and building research and knowledge sharing networks.

Recently, the burden of disease in low and medium human development index (HDI) countries has shifted from being dominated by infectious, maternal, neonatal and nutritional diseases to being dominated by non-communicable diseases (NCDs) [4]. Global Burden of Disease Study (GBD) 2019 showed that stroke was the third leading cause of death and disability (measured by disability-adjusted life years [DALYs], 5.7 % of total DALYs) and the second-leading cause of death (11.6 % of total deaths) worldwide [5]. More than 75 % of stroke deaths and 80 % of DALYs occur in low-income and middle-income countries. Previous studies have found that the mean global lifetime risk of stroke increased from 22.8 % in 1990 to 24.9 % in 2016, in addition, the lifetime risk of stroke varies geographically, with the highest risks in East Asia, Central Europe and Eastern Europe [6]. Without immediate implementation of effective primary prevention strategies, the burden of stroke is likely to continue to increase. “B&R” member states also face the threat of stroke to varying degrees. An internally consistent and comparable assessment of incidence, mortality, and disease burden and trends of stroke for China and its partner countries will be paramount for the HSR initiative’s success. However, despite global stroke burden has been widely reported, little is known about the status and attainment of stroke by 66 countries from the BRI. In addition, the “B&R” region encompasses a vast spectrum of economic development levels and epidemiological profiles, ranging from aging societies in Eastern Europe to rapidly developing nations in Southeast Asia. This diversity creates a complex landscape for stroke prevention and management.

The GBD 2021 framework, with broad collection of data sources and statistical modeling approaches, enables the comparable assessment of stroke burden in terms of incidence, mortality, years lived with disability (YLDs), and disability-adjusted life years (DALYs). In this study, we aimed to compare the burden of stroke and its spatiotemporal trends from 1990 to 2021, and further explore the changing trend of YLDs stratified by gender and age in the member countries of the “B&R” to provide a basis for formulating stroke prevention and control policies for building a healthy “B&R”.

## Methods

### Data sources and definitions

We conducted a secondary analysis using publicly available data from the GBD 2021. GBD 2021 presented, for the first time, estimates of global health loss due to the COVID-19 pandemic, which estimated epidemiological quantity of interest for 288 causes of death by age-sex-location-year for 25 age groups (from birth to 95 years and older), sex (males, females, and both sexes combined), location (204 countries and territories grouped into 21 regions and seven super-regions), and every year from 1990 to 2021(7, 8). GBD 2021 employed a unified framework to calculate excess mortality due to the COVID-19 pandemic while simultaneously generating cause-specific estimates for non-COVID-19 diseases including stroke. COVID-19 was modeled as a competing cause in the all-cause mortality rather than being directly subtracted from stroke estimates. We adopted standard epidemiological measures, such as incidence and mortality, as well as summary measure of health (YLDs, and DALYs) due to stroke. Age-standardized rates for incidence, mortality, YLDs and DALYs were computed with a global age structure from 2021. YLDs measure the amount of time people lose to diseases and injuries that reduce their health but do not cause death. YLDs was estimated by multiplying stroke prevalence with the corresponding disability weight. DALYs are a comprehensive indicator to assess the disease burden of disability and premature death. DALYs are equal to YLDs plus years of life lost (YLLs). YLLs are calculated as the product of counts of deaths caused by stroke and a standard remaining life expectancy at the age of death. The age-standardized rates were corrected using the direct method and the world standard population to account for differences in the age structure of the population. To ensure transparency and replicability, our study follows the Guidelines for Accurate and Transparent Health Estimates Reporting (GATHER) [7]. All data for this study were obtained from the Institute for Health Metrics and Evaluation (IHME) website.

In this study, “B&R” initiative refers to an organization of cooperation in political, economic, cultural and other related fields, including 66 member states. The 66 member countries are divided as follows [1]: East Asia: China [2]; Central Asia: Armenia, Azerbaijan, Georgia, Kazakhstan, Kyrgyzstan, Mongolia, Tajikistan, Turkmenistan, Uzbekistan [3]; South Asia: Bangladesh, Bhutan, India, Nepal, Pakistan [4]; Southeast Asia: Cambodia, Indonesia, Laos, Malaysia, Maldives, Burma, Philippines, Sri Lanka, Thailand, Vietnam [5]; High-income Asia pacific: Brunei, Singapore [6]; North Africa and Middle East: Afghanistan, Bahrain, Egypt, Iran, Iraq, Jordan, Kuwait, Lebanon, Oman, Palestine, Qatar, Saudi Arabia, Syria, Turkey, United Arab Emirates, Yemen [8]; Central Europe: Albania, Bosnia and Herzegovina, Bulgaria, Croatia, Czechia, Hungary, Montenegro, North Macedonia, Poland, Romania, Serbia, Slovakia, Slovenia [9]; Eastern Europe: Belarus, Estonia, Latvia, Lithuania, Republic of Moldova, Russia, Ukraine [7]; Western Europe: Cyprus, Greece, Israel. The division of the “B&R” countries is based on the division of global regions and international political and economic organizations in GBD.

### Statistical analyses

We computed absolute numbers and age-standardized rates of incidence, mortality, YLDs and DALYs to quantify the burden of stroke, grouped by gender, age and three major risk factors in “B&R” countries. Age-standardized estimates allow comparisons across time, countries and subregions, and are adjusted for differences in the age distribution of the population. For each estimated metrics, the 95 % uncertainty interval (UI) were reported. 95 % UI was calculated by taking 1,000 draws from the posterior distribution of each quantity, using the 2.5th and 97.5th ordered draw of the uncertainty distribution [5]. Age was divided into four groups: less than 15 years, 15–49 years, 50–74 years and ≥75 years. Trends of disease burden from 1990 to 2021 were evaluated using average annual percent change (AAPC), which were calculated by the Joinpoint Regression Program (Version 4.9.0.0. March 2021) [10]. A linear regression model was fitted on the natural logarithm of age-standardized rates: Y=β_0_+β_1_year+ε, where Y denotes the log-transformed rate, β_0_ is the intercept, β_1_ reflects the positive or negative temporal change, year represents the calendar year, and ε is the error term assumed to be independently and identically distributed with constant variance. The optimal number and location of joinpoints were determined through an iterative algorithm minimizing the Bayesian Information Criterion (BIC), with statistical significance evaluated using a Monte Carlo permutation test (4,999 permutations, α=0.05), allowing a maximum of five joinpoints. For each segment, the annual percent change (APC) was computed as 100 × (exp(β_1_)-1), with its 95 % confidence interval (CI) derived from the CI of β_1_. The AAPC was calculated as 100 × (exp(β_avg_)-1), where β_avg_ is the length-weighted average slope, with its 95 %CI calculated accordingly. An upward trend was considered if both the AAPC estimate and the lower bound of its 95 %CI were greater than zero. Conversely, a downward trend was identified when both the AAPC and the upper bound of the 95 %CI were less than zero. Additionally, we explored the long-term trends of stroke age-standardized YLDs rate attributable to Level 1 risk factors (behavioral, environmental/occupational and metabolic risks) from 1990 to 2021 in “B&R” countries. All analysis was conducted using the Joinpoint Regression Program (Version 4.9.0.0. March 2021). p<0.05 was considered statistically significant.

## Results

Absolute number of incidences, mortality, YLDs and DALYs in 2021 caused by stroke in each member country of the “B&R” are shown in Table 1. We noted that there were significant geographic differences in number of stroke incidence, mortality, YLDs and DALYs across countries, with China, India and Indonesia being the three countries with the highest burden of stroke. In 2021, there were 4.09 million (95 % UI 3.59 to 4.70) incident stroke, 2.59 million (95 % UI 2.18 to 3.03) deaths, 5.09 million (95 % UI 3.62 to 6.59) YLDs and 53.19 million (95 % UI 45.11 to 61.96) DALYs due to stroke in China. The country with the lowest number of incidence and DALYs for stroke is the Maldives in Southeast Asia (464.79, 95 % UI: 427.53–506.54 and 5,568.81, 95 % UI: 4,659.53–6,490.95).

**Table 1:** Absolute number of incidence, mortality, YLDs and DALYs due to stroke of the “B&R” countries in 2021.

	Incidence		Mortality		YLDs		DALYs	
	Number	95 %UI	Number	95 %UI	Number	95 %UI	Number	95 %UI
Global	11,946,273.94	10,772,079.55, 13,219,841.45	7,252,675.54	6,566,884.21, 7,808,179.59	15,210,424.48	10,985,667.17, 19,424,864.47	160,457,220.63	147,781,420.22, 171,642,573.58
**SDI regional**								
High	1,800,428.33	1,632,214.75, 1,980,662.42	797,759.98	683,006.24, 860,185.13	3,164,559.47	2,287,764.59, 4,028,220.18	15,221,301.66	13,730,362.47, 16,390,093.07
High-middle	3,093,653.17	2,748,262.04, 3,479,810.77	1,942,073.51	1,725,736.08, 2,137,663.38	3,874,790.97	2,806,644.53, 4,957,698.83	38,405,062.48	34,661,957.72, 42,299,983.58
Middle	4,214,749.73	3,795,042.43, 4,706,675.78	2,681,027.20	2,384,098.45, 2,945,687.77	5,078,663.69	3,651,104.17, 6,519,975.99	59,875,244.37	54,005,592.32, 65,174,884.76
Low-middle	2,028,593.91	1,854,695.95, 2,209,351.25	1,349,069.90	1,239,586.45, 1,454,472.00	2,193,208.29	1,596,706.89, 2,773,753.14	33,704,922.88	30,994,996.83, 36,497,559.37
Low	798,767.07	736,933.48, 865,563.31	475,825.81	424,761.16, 528,085.27	887,595.22	651,690.70, 1,122,252.30	13,104,740.67	1,1,571,852.47, 14,674,519.98
**East Asia**								
China	4,090,480.12	3,593,818.73, 4,699,827.92	2,591,646.92	2,179,395.77, 3,032,694.92	5,089,856.76	3,624,862.57, 6,591,427.31	53,190,691.17	45,108,715.79, 61,958,023.94
**Central Asia**								
Armenia	4,614.17	4,223.16, 5,045.66	2,810.28	2,466.86, 3,137.51	7,486.50	5,381.80, 9,426.98	55,853.80	50,271.03, 62,316.81
Azerbaijan	17,259.98	15,879.79, 18,708.67	8,003.98	6,592.18, 9,574.95	20,081.02	14,397.72, 25,513.60	186,713.95	152,819.57, 226,338.29
Georgia	12,565.88	11,536.14, 13,591.05	10,283.81	9,121.32, 11,411.65	12,545.72	9,123.38, 15,892.18	184,348.68	163,698.26, 204,771.65
Kazakhstan	37,266.57	33,934.13, 40,644.41	22,729.23	19,890.44, 25,823.01	50,102.86	36,023.56, 63,470.10	508,613.11	441,060.61, 576,135.13
Kyrgyzstan	7,805.56	7,129.16, 8,557.92	3,942.58	3,327.88, 4,584.16	10,146.52	7,258.67, 12,935.49	108,093.19	91,881.98, 125,773.16
Mongolia	5,513.64	5,165.84, 5,881.02	2,561.07	2093.48, 3,063.36	6,450.81	4,608.47, 8,200.40	72,129.30	60,014.01, 85,589.23
Tajikistan	11,636.46	10,686.98, 12,667.54	5,574.05	4,427.09, 6,710.31	12,955.71	9,270.49, 16,590.29	141,135.20	112,359.12, 170,135.18
Turkmenistan	9,318.23	8,535.67, 10,158.60	5,758.61	4,534.49, 7,015.73	12,703.45	9,119.06, 16,275.73	158,046.16	126,096.98, 192,736.74
Uzbekistan	59,953.46	55,241.67, 65,435.23	22,174.79	19,132.73, 25,699.87	70,820.50	51,008.92, 90,104.72	580,744.20	505,374.64, 671,586.88
**South Asia**								
Bangladesh	221,157.12	203,556.48, 240,970.43	176,877.39	143,884.85, 215,202.16	208,406.98	152,038.39, 263,604.17	3,941,837.97	3,208,630.56, 4,819,419.31
Bhutan	666.07	607.47, 726.49	394.45	312.11, 484.65	742.36	535.88, 945.37	8,838.76	6,973.95, 10,895.70
India	1,250,948.79	1,127,370.79, 1,378,326.87	772,975.60	695,375.75, 857,759.24	1,315,776.79	958,132.71, 1,675,608.91	19,436,100.81	17,539,463.62, 21,385,220.07
Nepal	26,152.42	23,760.06, 28,481.40	17,252.16	13,817.33, 21,818.80	25,849.33	18,917.77, 32,726.33	411,354.13	329,736.89, 519,570.36
Pakistan	198,179.94	179,699.32, 218,781.01	99,759.79	82,952.14, 123,652.59	226,026.44	163,661.89, 288,259.93	2,803,526.09	2,318,364.00, 3,459,374.22
**Southeast Asia**								
Cambodia	23,554.98	21,697.65, 25,455.62	17,903.03	14,033.24, 21,561.12	27,331.64	19,442.97, 35,262.08	435,171.56	339,039.74, 534,641.84
Indonesia	543,349.05	486,499.93, 611,194.52	404,559.36	338,452.95, 464,309.25	740,782.33	532,919.35, 954,773.93	10,624,061.61	8,956,510.04, 12,309,256.95
Laos	9,431.62	8,678.20, 10,198.83	7,087.80	5,665.57, 8,753.48	12,286.89	8,828.28, 15,743.79	192,841.49	153,953.20, 240,429.48
Malaysia	44,739.34	40,576.49, 49,456.21	23,245.51	21,070.11, 25,703.94	75,409.32	54,619.35, 95,363.27	593,439.48	541,123.44, 650,112.83
Maldives	464.79	427.53, 506.54	224.64	184.29, 261.63	786.86	566.89, 1,001.23	5,568.81	4,659.53, 6,490.95
Burma	88,388.84	81,815.29, 95,547.19	77,214.74	61,936.36, 95,593.66	112,669.46	80,375.72, 143,528.42	1,961,453.05	1,593,525.40, 2,424,347.13
Philippines	132,877.13	120,545.57, 147,795.13	83,819.39	71,373.57, 96,157.98	196,904.36	141,311.99, 251,839.08	2,364,482.04	2,036,638.89, 2,715,266.93
Sri Lanka	31,012.53	27,768.23, 34,183.64	25,181.48	17,812.98, 32,866.49	51,287.24	37,174.10, 64,978.93	498,162.19	360,600.47, 642,281.21
Thailand	117,556.87	107,504.42, 127,943.76	69,976.16	54,311.60, 86,359.26	208,558.97	150,880.21, 264,323.64	1,676,447.81	1,346,657.44, 2,042,438.94
Vietnam	195,806.07	183,222.40, 209,488.60	166,954.18	139,507.69, 193,350.86	241,891.81	174,668.40, 309,600.44	3,768,750.61	3,131,538.11, 4,444,248.36
**High-income Asia pacific**								
Brunei	509.11	457.41, 562.10	170.40	147.19, 197.86	810.01	589.78, 1,021.74	5,029.48	4,389.79, 5,831.54
Singapore	6,543.76	5,807.35, 7,439.17	1,173.67	1,015.69, 1,279.81	13,868.90	10,067.70, 17,638.61	35,659.33	31,473.77, 39,596.51
**North Africa and middle East**								
Afghanistan	21,823.81	20,091.21, 23,672.65	15,126.22	11,498.04, 18,967.79	22,829.25	16,851.01, 28,893.62	459,365.88	354,787.37, 579,285.18
Bahrain	725.97	650.40, 809.81	358.67	305.67, 422.73	1,324.97	985.61, 1,663.52	10,050.31	8,585.76, 11,622.50
Egypt	104,553.80	94,514.48, 117,362.47	73,354.88	60,170.80, 88,761.26	121,551.08	88,284.39, 155,296.80	1,847,903.26	1,511,674.94, 2,234,735.78
Iran	76,271.63	67,431.43, 86,132.34	41,908.22	37,515.95, 45,630.12	108,041.83	78,674.55, 137,730.26	904,630.42	828,258.53, 981,197.99
Iraq	45,127.00	40,965.90, 49,752.70	30,848.28	24,447.93, 37,085.72	51,034.52	37,257.00, 64,854.29	764,342.80	601,922.98, 931,775.76
Jordan	11,792.64	10,576.05, 13,006.61	3,602.78	2,905.00, 4,357.01	16,064.46	11,815.59, 20,349.88	96,554.84	81,033.45, 113,907.20
Kuwait	3,331.54	3,029.61, 3,664.32	768.78	632.80, 921.39	5,798.76	4,221.51, 7,411.76	23,265.62	19,791.32, 27,025.41
Lebanon	7,119.76	6,409.13, 7,850.51	2,957.29	2,424.88, 3,475.52	8,972.70	6,610.67, 11,330.29	57,223.15	49,413.87, 65,895.25
Oman	3,281.99	3,011.77, 3,580.58	1,008.32	814.04, 1,202.27	5,273.69	3,811.02, 6,755.16	30,123.89	24,921.34, 35,404.25
Palestine	3,661.93	3,361.97, 4,013.44	1,983.04	1,712.38, 2,250.39	3,990.93	2,903.11, 5,057.01	46,449.11	40,510.66, 52,131.79
Qatar	1,456.82	1,278.95, 1,666.48	249.65	192.90, 318.53	2,871.52	2,126.65, 3,637.64	10,139.13	8,196.84, 12,284.78
Saudi Arabia	28,005.84	25,498.69, 30,595.76	13,437.98	10,837.61, 16,685.38	36,818.46	26,803.67, 46,999.81	439,094.07	351,301.79, 545,201.06
Syria	16,059.51	14,652.93, 17,592.07	10,540.54	8,134.91, 13,305.16	20,079.13	14,750.00, 25,594.63	260,135.24	201,808.89, 331,674.34
Turkey	97,866.22	87,326.13, 108,254.68	59,380.96	49,029.36, 70,649.39	131,055.94	95,496.97, 167,133.97	1,185,230.67	995,753.04, 1,388,143.19
United Arab Emirates	10,210.14	8,910.53, 11,682.84	1,243.55	970.54, 1,518.69	14,591.94	10,628.52, 18,568.55	51,749.58	42,508.47, 61,938.04
Yemen	24,090.76	22,029.67, 26,109.52	18,483.07	13,793.24, 24,848.65	25,539.50	18,820.25, 32,168.12	485,214.26	364,187.10, 635,177.87
**Central Europe**								
Albania	6,251.71	5,760.28, 6,777.49	6,157.39	4,998.07, 7,449.05	5,806.38	4,188.03, 7,404.31	97,119.81	79,011.26, 115,879.57
Bosnia and Herzegovina	10,116.76	8,941.06, 11,281.63	7,097.57	5,678.36, 8,363.01	12,647.81	9,218.85, 16,120.49	126,649.39	102,834.79, 147,781.21
Bulgaria	31,161.71	27,887.61, 34,241.19	28,016.92	24,547.17, 31,556.39	28,705.01	21,002.40, 36,586.76	483,790.67	424,450.64, 550,902.46
Croatia	11,027.13	10,421.63, 11,635.93	6,675.39	5,838.16, 7,519.14	13,289.98	9,692.01, 16,782.45	110,342.20	98,118.05, 123,357.63
Czechia	23,180.99	20,453.02, 26,073.78	9,548.87	8,357.56, 10,703.71	33,980.31	24,740.96, 42,900.98	176,786.47	156,309.28, 196,800.08
Hungary	22,139.75	19,917.05, 24,704.01	11,845.81	10,249.01, 13,397.31	29,509.62	21,349.86, 37,615.30	222,897.42	195,131.04, 250,742.66
Montenegro	1,813.82	1,686.34, 1,959.01	1,907.79	1,643.65, 2,197.66	1,427.73	1,039.38, 1,807.56	31,861.57	27,281.89, 37,180.66
North Macedonia	7,486.79	6,722.56, 8,279.19	6,605.89	5,437.65, 7,819.62	7,098.35	5,057.58, 9,034.08	119,595.89	97,698.17, 141,508.01
Poland	72,341.36	62,221.59, 82,838.23	45,065.40	39,718.53, 49,204.87	89,657.08	63,101.29, 115,299.10	800,348.74	725,844.69, 875,975.21
Romania	63,903.54	57,646.57, 69,845.21	52,999.54	47,064.40, 58,786.40	67,991.03	49,469.49, 86,170.44	903,023.21	809,849.51, 999,176.56
Serbia	31,800.05	28,992.42, 34,873.99	27,994.99	23,763.19, 32,519.38	27,473.19	19,678.49, 34,617.66	458,173.15	388,356.02, 531,939.11
Slovakia	12,675.61	11,164.69, 14,309.49	6,311.76	5,310.24, 7,452.04	19,682.02	14,330.38, 24,834.33	126,684.57	108,242.96, 147,139.62
Slovenia	3,427.44	3,119.52, 3,746.32	1,847.69	1,555.48, 2,076.62	4,483.11	3,252.08, 5,689.49	28,777.85	25,099.97, 32,121.20
**Eastern Europe**								
Belarus	27,034.13	23,943.22, 30,070.73	15,977.46	13,414.02, 18,666.50	32,197.61	23,156.32, 40,885.14	321,957.56	270,374.41, 376,324.61
Estonia	2,284.99	2030.66, 2,562.68	1,148.72	987.73, 1,290.49	3,238.43	2,360.29, 4,121.07	20,521.47	18,019.92, 22,973.56
Latvia	6,603.02	5,974.73, 7,281.80	5,051.50	4,430.71, 5,578.11	7,185.99	5,211.36, 9,147.43	81,238.98	72,187.20, 89,558.71
Lithuania	10,056.35	8,818.96, 11,377.73	4,627.53	4,104.55, 5,107.28	9,146.67	6,427.91, 11,909.48	79,174.46	69,944.62, 87,236.63
Republic of Moldova	8,583.50	7,692.62, 9,500.95	5,144.49	4,616.31, 5,688.26	9,562.44	6,943.25, 12,170.06	111,871.52	101,630.27, 123,501.55
Russia	421,513.91	368,384.81, 480,718.39	311,447.30	284,885.21, 335,147.94	443,432.34	321,470.63, 567,251.60	5,892,124.66	5,460,053.67, 6,338,953.13
Ukraine	134,283.50	116,189.33, 153,495.31	82,352.46	64,482.74, 102,151.98	152,587.33	110,791.65, 198,677.14	1,631,824.26	1,276,149.51, 2,022,861.43
**Western Europe**								
Cyprus	1,260.95	1,160.78, 1,370.74	770.11	639.27, 904.96	1,368.47	983.84, 1,748.54	12,328.76	10,470.28, 14,160.64
Greece	26,949.97	24,532.44, 29,496.22	17,206.97	14,663.33, 18,576.04	27,248.18	20,169.94, 34,425.98	247,366.79	220,650.09, 265,854.69
Israel	7,943.11	7,104.19, 8,895.65	2,837.29	2,399.27, 3,118.97	13,215.44	9,612.57, 16,732.60	54,340.41	48,266.80, 59,347.02

Data are presented as point estimates with 95 % uncertainty intervals; DALYs, disability-adjusted life years; YLDs, years lived with disability; UI, uncertainty interval.

**Figure 1:**
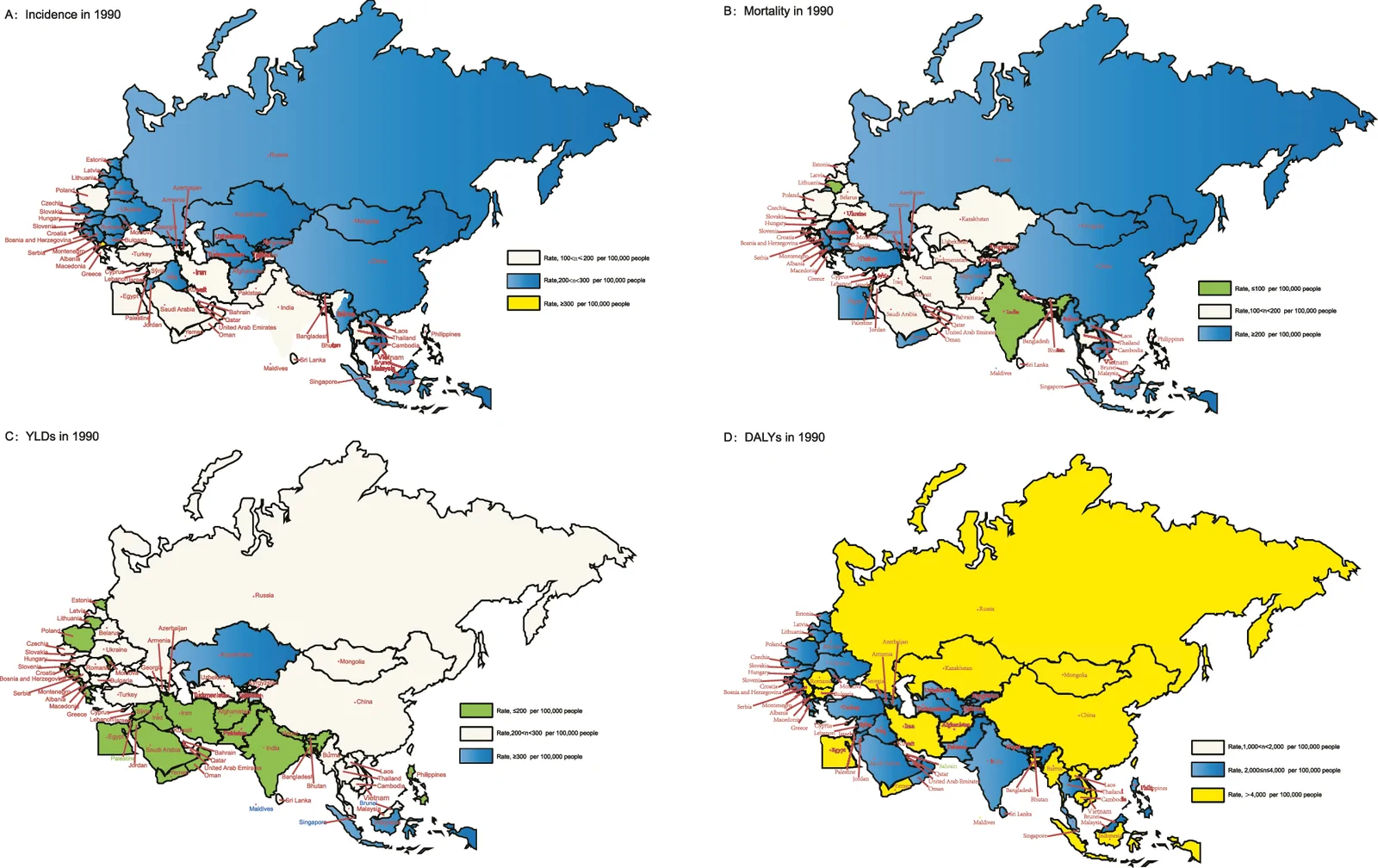
The age-standardized rates of incidence, mortality, YLDs and DALYs due to stroke in 1990 for “the Belt & Road” countries (A) age-standardized incidence rate in 1990 (B) age-standardized mortality rate in 1990 (C) age-standardized YLDs rate in 1990 (D) age-standardized DALYs rate in 1990.

**Figure 2:**
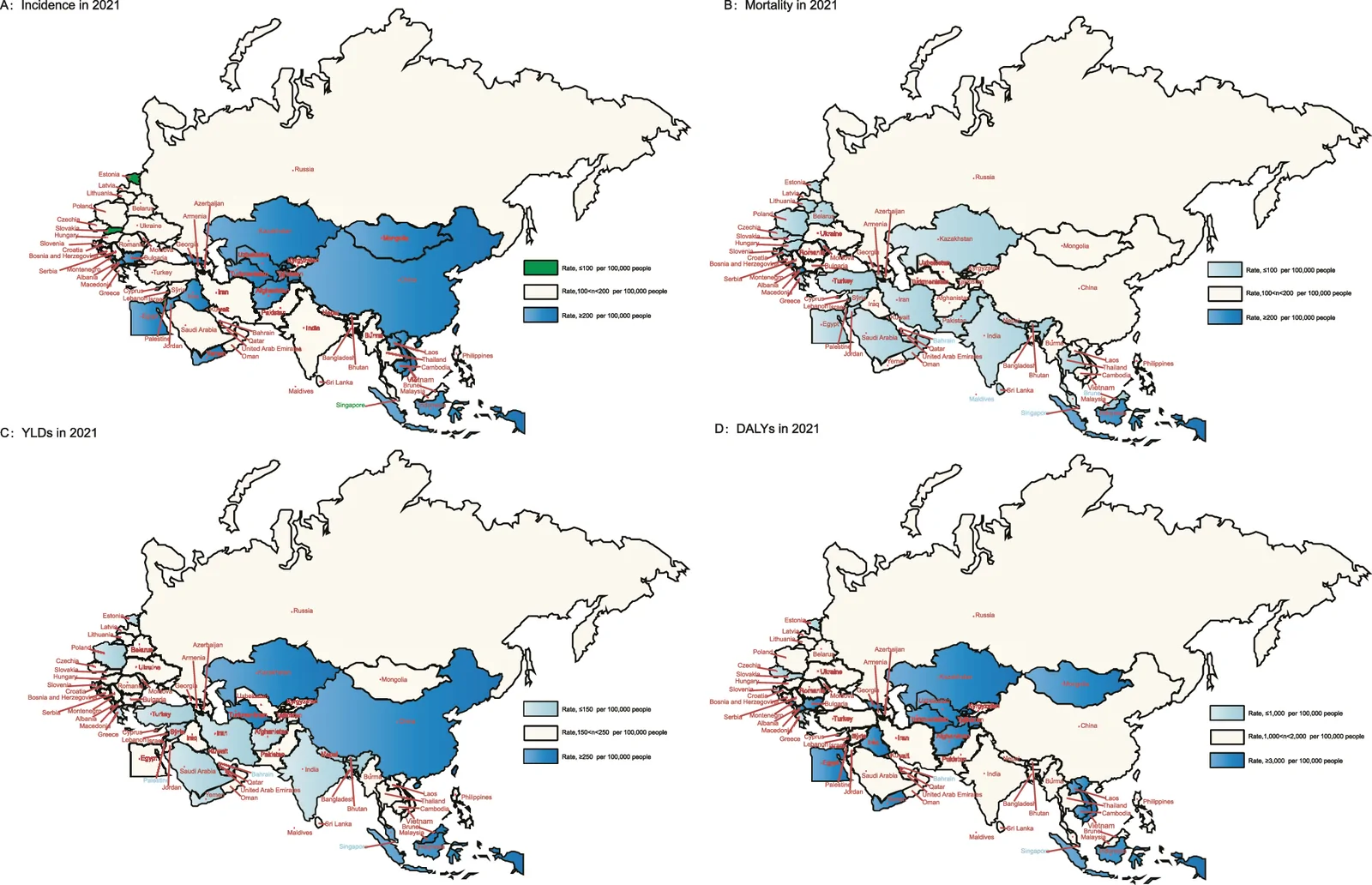
The age-standardized rates of incidence, mortality, YLDs and DALYs due to stroke in 2021 for “the Belt & Road” countries (A) age-standardized incidence rate in 2021 (B) age-standardized mortality rate in 2021 (C) age-standardized YLDs rate in 2021 (D) age-standardized DALYs rate in 2021.

Figures 1 and 2 intuitively showed the age-standardized rates of incidence, mortality, YLDs and DALYs due to stroke in 1990 and 2021 in member countries of the “Belt and Road” initiative. From 1990 to 2021, the age-standardized incidence, mortality, YLDs and DALYs of stroke were generally declining in most member States. In 2021, the country with the highest age-standardized YLDs rate of stroke was Indonesia (291.21 per 100,000), followed by Turkmenistan (288.21 per 100,000). The Israel enjoyed the lowest rates of incidence and DALYs, respectively (65.13 per 100,000 and 426.46 per 100,000, respectively). In the high-income Asia-Pacific region, age-standardized incidence, mortality, YLDs and DALYs due to stroke declined rapidly in Singapore and Brunei from 1990 to 2021, for example, stroke deaths in Singapore fell rapidly from 90.58 per 100,000 people in 1990 to 14.23 per 100,000 people in 2021. See Supplementary Table S1 for more details.

**Figure 3:**
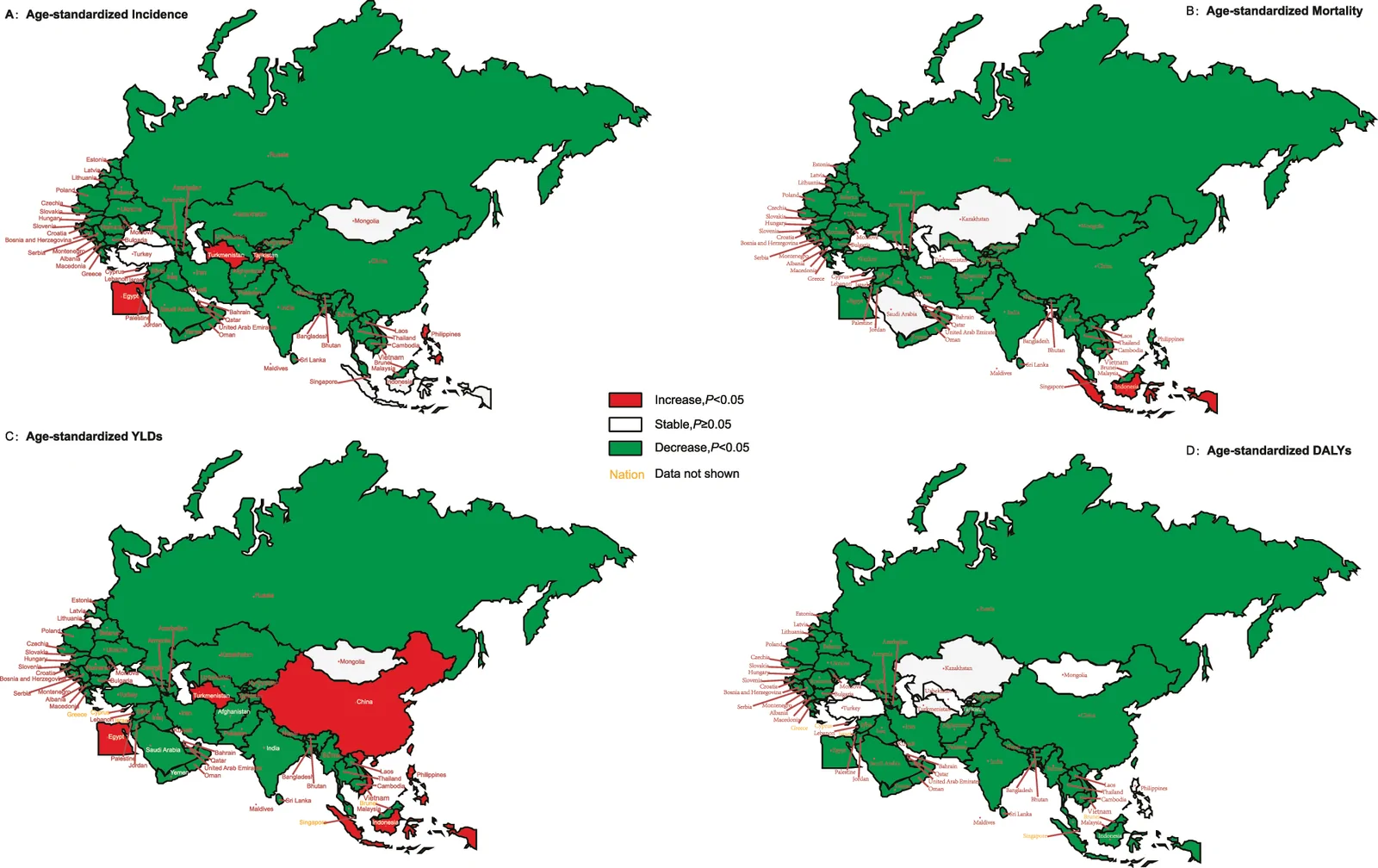
The trends of age-standardized rates of incidence, mortality, YLDs and DALYs due to stroke for 1990–2021 for “the Belt & Road” countries (A) the AAPC of age-standardized incidence rate (B) the AAPC of age-standardized mortality rate (C) the AAPC of age-standardized YLDs rate (D) the AAPC of age-standardized DALYs rate.

The average annual percentage change (AAPC) of age-standardized rates of incidence, mortality, YLDs and DALYs due to stroke for 1990–2021 for “the Belt & Road” countries was displayed in Figure 3. From 1990 to 2021, the AAPC of age-standardized incidence, mortality and DALYs from stroke in China decreased by 0.37 % (95 %CI: −0.44 % to −0.29 %, p<0.001), 1.79 % (95 %CI: −2.01 % to −1.57 %, p<0.001) and 1.93 % (95 %CI: −2.09 % to −1.77 %, p<0.001), respectively. The AAPC values of incidence, mortality, YLDs and DALYs in “B&R” European countries showed a decreasing trend, except for age-standardized mortality in Montenegro and North Macedonia, as well as age-standardized DALYs rate in Montenegro. In terms of age-standardized incidence, we found no statistically significant difference in AAPC for Mongolia and Indonesia. Only Georgia, Kazakhstan, Turkmenistan, Kuwait, United Arab Emirates, Montenegro and North Macedonia were found to have no statistically significant differences in AAPC for age-standardized mortality. The AAPC of age-standardized incidence showed an increasing trend in Azerbaijan, Tajikistan, Turkmenistan, Uzbekistan, Philippines and Egypt. For age-standardized YLDs rate, China, Turkmenistan, Philippines, Viet Nam and Egypt showed an increasing trend. See Supplementary Table S2 for more details.

**Figure 4:**
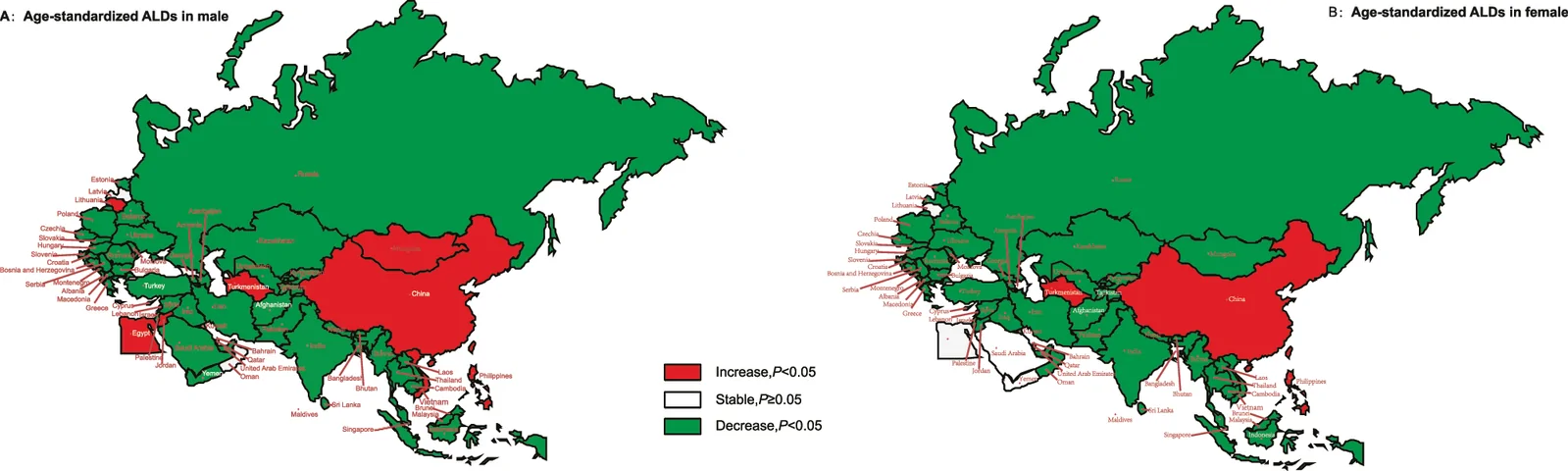
Visualization of the trends of age-standardized YLDs rate due to stroke, stratified by gender, for 1990–2021 for “the Belt & Road” countries (A) the AAPC of age-standardized YLDs rate in male (B) the AAPC of age-standardized YLDs rate in female.

Figure 4 illustrated the AAPC values of age-standardized YLDs rate in each member country of the “B&R” in males and females. Most of countries in the “B&R” countries showed a downward trend in AAPC, however, with an upward trend for both sexes in China, Turkmenistan and Philippines. Turkmenistan had the highest increase in AAPCs of age-standardized YLDs from 1990 to 2021 for men (AAPC=0.60 %, 95 %CI: 0.58–0.61 %), while for women Philippines had the highest increase (AAPC=0.57 %, 95 %CI: 0.55–0.59 %). For men, the AAPC in Georgia, Lebanon, Oman and Latvia were stable between 1990 and 2021, while the change trend of YLDs was stable for female in Egypt and Lithuania (p≥0.05) (Supplementary Table S3).

**Figure 5:**
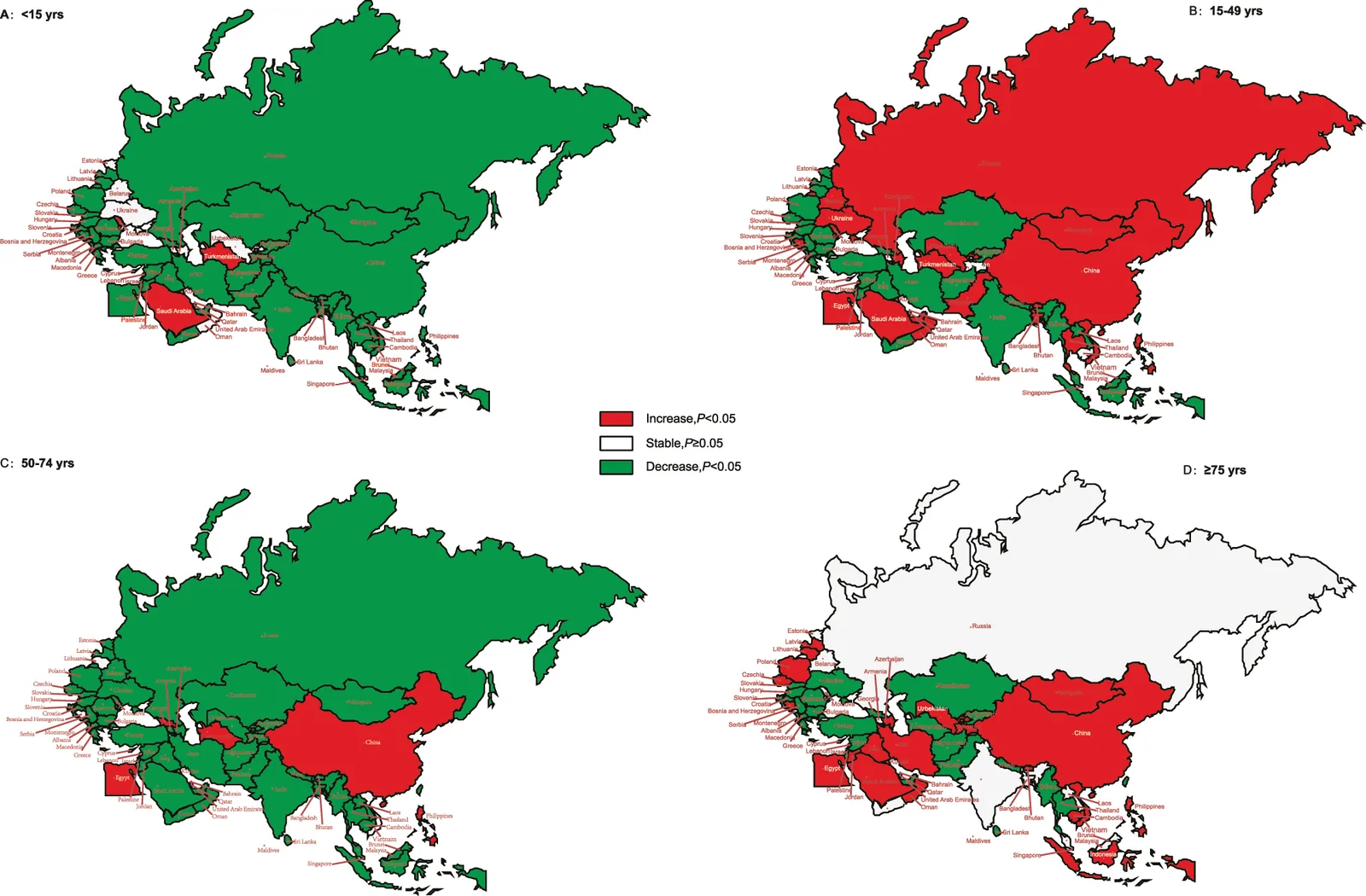
Visualization of the trends of YLDs rate due to stroke, stratified by age for 1990–2021 for “the Belt & Road” countries (A) YLDs rate in people aged <15 yrs (B) YLDs rate in people aged 15–49 yrs (C) YLDs rate in people aged 50–74 yrs (D) YLDs rate in people aged ≥75 yrs.

Figure 5 showed the long-term trends of YLDs rate due to stroke, stratified by age for 1990–2021 for the “B&R” countries. YLDs in people over 75 years of age exhibited an upward trend from 1990 to 2021, with AAPC values of 0.15 % (95 %CI: 0.12–0.17 %), while the other three age groups showed downward trends. For the 15–49 and 50–74 age groups, the decreasing trends in YLDs were observed across SDI regions, with the highest decrease in AAPC in the Low SDI region (0.42 % and 0.54 %, respectively). For under 15 years of age, YLDs due to stroke in most member states showed a downward trend, while increasing trends were noted in Saudi Arabia and Turkmenistan. For people aged 15–49 years, YLDs for stroke showed an increasing trend in 22 member states, with the highest in Turkmenistan (AAPC=1.23, 95 %CI: 1.20–1.27 %). For people aged 50–74 years, Philippines in Southeast Asia had the greatest AAPC value. See Supplementary Table S4 for more details.

**Figure 6:**
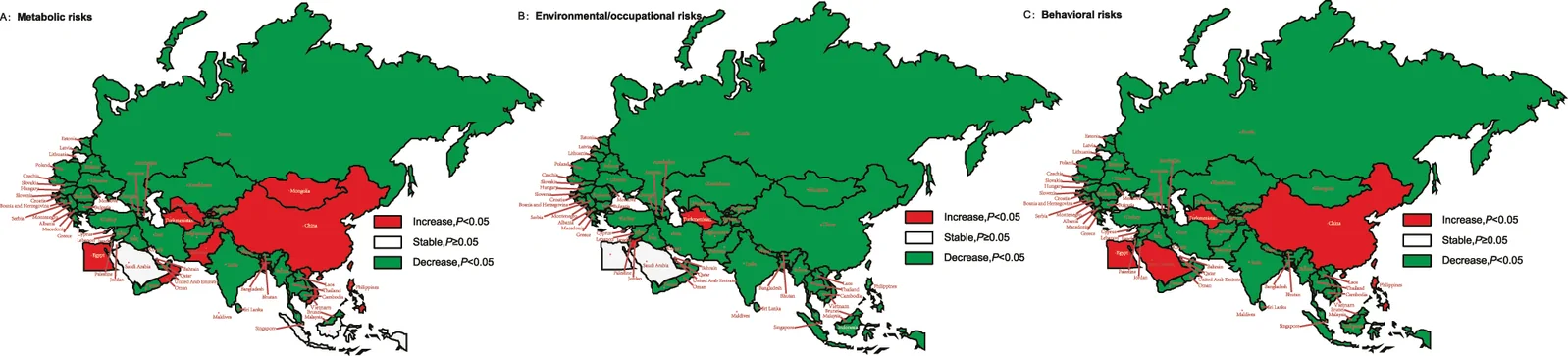
The long-term trends of stroke YLDs rate attributable to risk factors from 1990 to 2021 for “the Belt & Road” countries.

Figure 6 illustrated the long-term trends of stroke age-standardized YLDs rate attributable to risk factors from 1990 to 2021. We focused on three major risk factors for stroke: behavioral, environmental/occupational and metabolic risks. There was a significant decline in the age-standardized YLDs rate for the three risk factors, with AAPCs of −0.12 % (95%CI, −0.13 % to −0.11 %), −0.92 % (95 %CI, −1.01 % to −0.83 %) and −0.53 % (95 %CI, −0.55 % to −0.51 %), respectively. High-SDI regions exhibited a more pronounced decline in age-standardized YLDs rate, whereas Middle SDI regions showed a less significant decline for behavioral risks. At the country level, China stood out with a significant increase in YLDs rate for metabolic risks (AAPC=0.83 %, 95 %CI, 0.79 % to 0.88 %) compared with the global trend, however, Singapore showed a significant decline (AAPC=−2.62 %, 95 %CI, −2.71 % to −2.52 %). Turkmenistan had the largest AAPC in the age-standardized YLDs rate of stroke attributed to environmental/occupational risks. See Supplementary Table S5 for more details.

## Discussion

In the present study, we analyzed the disease burden and its trends of stroke over a 30-year period from 1990 to 2021 in the member states of the “B&R”. The results showed that different member states were at risk of stroke to varying degrees, with China, India and Indonesia in the Asian region being the three countries with the highest absolute numbers of stroke. According to the GBD 2019 study, in absolute terms, the global incidence of stroke has increased by 70 %, its mortality increased by 43 %, stroke-induced DALYs increased by 32 %, and the burden of stroke has increased more in low- and middle-income countries (LMICs) than in high-income countries (HICs) over the past 30 years [11]. As the two largest developing countries by population, China and India accounted for 36.3 % of the world’s population in 2019 and 19.0 % of the cumulative global gross domestic product in 2018, with significant impact on NCDs. Studies have found a strong negative relationship between stroke and socioeconomic status, due to inadequate post-stroke care among low-income populations [12]. The increased absolute numbers of stroke in developing countries may be due to inadequate health care services, lack of awareness of stroke and its risk factors, lack of economic resources and good primary preventive health care systems, and lack of emergency services and facilities for acute stroke management [13]. In addition, a large percentage of people in developing countries live in rural areas where healthcare is not accessible. Therefore, stroke prevention strategies in LMICs should differ from those adopted in HICs, the Health Belt and Road Initiative provide new solutions, such as strengthening regional scientific and technological cooperation through targeted, resource-sharing means, establishing joint laboratories or research centers, and transferring international technology and knowledge.

Findings from this study found that the age-standardized incidence, mortality, YLDs and DALYs of stroke were generally declining in most “B&R” member states. Quanquan Ding et al. [14]. demonstrated that the global age-standardized incidence rate of ischemic stroke decreased from 1990 to 2019, with an estimated annual percentage change of −0.43 (95 % CI: −0.54 to −0.32), which was consistent with our results. From 1990 to 2019, age-standardized estimates of stroke decreased significantly, while absolute numbers increased. Longer life expectancy, lower premature death rates and aging populations may account for some of these findings. In the high-income Asia-Pacific region, age-standardized metrics due to stroke declined rapidly in Singapore and Brunei, however, Indonesia has the highest age-standardized incidence and YLDs of stroke. The differences in the burden of stroke disease in “B&R” member states may be related to the differences in the quality of stroke prevention care, rehabilitation treatment and the different distribution of risk factors in different countries. The results of GBD also depended on the consistency of government policy and the state of economic development to a large extent.

We found that the AAPC values of age-standardized incidence, mortality, YLDs and DALYs showed a downward trend in “B&R” European countries. Over the past three decades, many European countries have implemented population-level hypertension screening programs, expanded statin utilization, and enforced tobacco control policies, all of which are strongly associated with reduced stroke incidence. Improved access to acute stroke units, thrombolysis, and organized rehabilitation services has further reduced case fatality and post-stroke disability. Furthermore, our results showed that the AAPC of age-standardized incidence and YLDs rate showed an increasing trend in Turkmenistan, Philippines and Egypt. These countries are developing countries that faced some basic public health challenges, including problems related to the double burden of communicable and non-communicable diseases, limited scientific productivity, and lack of standardization of treatment. Study indicate that Egypt faces substantial shortages in neurology specialists and stroke unit infrastructure outside major urban centers such as Cairo and Alexandria, resulting in limited access to thrombolysis and secondary prevention programs for rural populations. Additionally, out-of-pocket health expenditure remains high, which may delay care-seeking and reduce adherence to antihypertensive therapy. These systemic constraints likely contribute to the observed upward trajectory in stroke-related disability. Philippines, as an archipelagic nation, faces persistent challenges in delivering specialized stroke care to rural and remote island communities, where access to diagnostic imaging and rehabilitation services is limited. Concurrently, dietary patterns have shifted toward processed foods and high-sodium diets, while tobacco use remains prevalent among males. Turkmenistan inherited the centralized medical system practiced by the Soviet Union and has carried out extensive health reforms [15]. One study found that Asia had 60 % of the world’s population, but only 20 % of the world’s neuroscientists, and the disparity in the distribution of medical resources was particularly pronounced in South and Southeast Asia [16]. This study could help research on stroke prevention and care and guide health-care resource allocation of different regions in “B&R” member countries.

Non-fatal health outcomes from diseases and injuries are a key consideration in promoting and monitoring the health of individuals and populations. We further explored the AAPC values of age-standardized YLDs rate by sex and age in each member country of the “B&R” in depth. The results showed that AAPC decreased in most of the countries along the “B&R”, but increased in China, Turkmenistan and Philippines for both sexes. This observed gender discrepancy may be related to physiological differences, lifestyle, substantial differences in the strength of association of stroke risk factors, as well as female-specific risk factors [17], 18]. Premenopausal estrogen confers vascular protection; postmenopausal decline accelerates atherosclerosis and elevates stroke risk. Pregnancy-associated hypertension and gestational diabetes confer long-term cardiovascular risk. In many B&R countries, males have higher rates of tobacco use and alcohol consumption, which elevate stroke incidence at younger ages. Conversely, females in several B&R regions, particularly in South and Southeast Asia, demonstrate lower physical activity levels and higher rates of untreated hypertension due to limited health-seeking autonomy. Meanwhile, we found that the age-standardized YLDs rate in Singapore showed a decreasing trend, and the decreasing amplitude of AAPC values increased with the increase of age. As life expectancy increases, people are likely to spend a greater number of years living with reduced health because the added years are at older ages with increased rates of disability. There were differences in age-specific standardized YLDs rates for stroke in different age groups in different “B&R” member countries. These variations also might be related to disparities in genetic vulnerability, lifestyle, dietary patterns and local environmental exposures among the different locations. For example, Mediterranean B&R countries may benefit from protective dietary patterns, whereas Central Asian and Southeast Asian nations face higher stroke risk associated with high-sodium and high-fat dietary transitions [19]. For under 15 years of age, childhood stroke is rare and GBD 2021 relies on sparse hospital-based registries, claims data, or verbal autopsy, rather than large-scale population-based surveillance, thus these estimates are associated with wider uncertainty intervals and greater model dependence, which should be interpreted cautiously. Regarding ≥75 age-standardization YLDs rate, age-standardization enables valid cross-population comparisons but does not eliminate absolute burden expansion from demographic shifts such as population aging and improved post-stroke survival, with residual trends potentially reflecting higher incidence or longer disability duration in the oldest-old. Additionally, improved diagnostic sensitivity and greater awareness of stroke in older adults may contribute to higher ascertainment over time.

The present study provides a comprehensive analysis of the age-standardized YLDs rates for stroke attributable to Level 1 risk categories (metabolic, environmental/occupational and behavioral risk). “B&R” countries span diverse epidemiological transitions, healthcare systems, and policy contexts. Level 1 categories allow for standardized, actionable comparisons across 66 countries, facilitating identification of which major modifiable risk categories drive inter-country disparities. We found that the significant decline in age-standardized YLDs rates for metabolic risks may be attributed to increased awareness, improved management of metabolic conditions and the implementation of effective public health policies. Metabolic risk factors at Level 1 as defined by the GBD framework, which aggregates multiple individual components such as high systolic blood pressure, high BMI, high fasting plasma glucose, and high LDL cholesterol. This aggregated approach does not allow us to determine which specific components are driving the observed trends. Moreover, not all metabolic risk components necessarily exhibit the same temporal trends. For instance, while high BMI and high FPG have generally increased across most “B&R” countries due to nutritional transitions and sedentary lifestyles, trends in high SBP and high LDL cholesterol may vary depending on the effectiveness of population-level hypertension screening and lipid-lowering interventions. Thus, future studies should examine component-specific trajectories to inform targeted interventions. We found that China showed a significant increase in age-standardized YLDs rate due to metabolic risks. This finding is at odds with the global trend and may be attributable to China’s accelerated economic growth, urbanization and alterations in dietary patterns [20], 21]. The observed increase underscores the need for targeted interventions that address the unique epidemiological transition occurring in China. Studies have found that Japan and South Korea have achieved significant reductions in stroke mortality and disability over the past three decades through aggressive population-level hypertension control, tobacco control policies, and access to acute stroke care under universal health coverage [22], 23]. In contrast, many “B&R” member countries-particularly in Central and South Asia and some in Southeast Asia – have made slower progress due to poor primary care infrastructure, low awareness of cardiovascular risk factors, and late adoption of prevention at the population level. Middle and low SDI regions require more fundamental interventions to reduce exposure to environmental and occupational risks.

Our analysis yields three core messages for policymakers and health practitioners under the Health Silk Road framework. First, stroke burden trends are highly heterogeneous across the B&R region, this divergence precludes one-size-fits-all intervention strategies; instead, tailored approaches reflecting national epidemiological profiles, health system capacity, and socioeconomic contexts are essential. For example, high-income members should sustain acute care and expand age-friendly rehabilitation, while low- and middle-income members need urgent investment in primary prevention infrastructure, population-level hypertension control, and salt reduction and tobacco policies. Second, sex-specific and age-specific disparities demand targeted prevention frameworks. Females in several B&R countries exhibit accelerating YLD trends driven by biological vulnerabilities, health system barriers, and social determinants that are not captured by gender-neutral policies. Older adults face expanding absolute burden despite age-standardized improvements, necessitating investment in age-friendly rehabilitation and long-term care infrastructure. Third, the COVID-19 pandemic has likely amplified pre-existing inequalities in stroke care access and outcomes. Post-pandemic recovery planning must prioritize resilient health systems capable of maintaining chronic disease management during public health emergencies, particularly in resource-constrained settings.

This study is based on the largest epidemiological dataset to date, and is the first to provide systematic estimates of stroke burden and trends, analyze the trend of YLDs, stratified by gender and age in “B&R” countries from 1990 to 2021. The main strength of this study is the collection of “B&R” wide epidemiological data using the same methods and modeling measures used in the GBD study. This study also has limitations. First, all limitations of the GBD methodology affect this study, which have been described previously [4], 24], 25]. In addition, GBD 2021 estimates reflect with-pandemic conditions. During 2020–2021, healthcare disruptions (reduced access to acute stroke care, delayed diagnosis, and interruption of cardiovascular risk factor management) may have increased stroke mortality and disability in certain regions. Changes in health-seeking behavior, physical activity, and medication adherence during lockdowns may also have affected stroke incidence and outcomes. Cause-of-death coding in some countries may have been influenced by the focus on COVID-19, potentially leading to misclassification of stroke-related deaths. These factors introduce uncertainty into the most recent trend estimates, and observed changes in 2020–2021 may partly reflect pandemic-related disruptions rather than true epidemiological transitions. Second, the weight of disability used to calculate YLDs may not be uniform at the demographic and sociodemographic levels. Moreover, we performed significance tests for AAPCs across dozens of countries, multiple indicators, and numerous subgroups without applying formal multiple comparison corrections (Bonferroni). While this approach aligns with standard conventions in GBD trend analyses, it could increase the risk of false-positive. Third, changes in the quality and completeness of available information can partly explain the differences found in comparing different “B&R” countries. Finally, we only analyzed stroke burden attributable to Level 1 risk categories and did not disaggregate these findings to Level 3 individual risk factors (e.g., high systolic blood pressure, smoking, ambient PM2.5 exposure), which limits the identification of country-specific intervention targets. Future research should incorporate Level 3 risk factor decomposition to inform more precise, tailored prevention strategies.

## Conclusions

This study compared the burden of stroke and its trends, and explored the changes of age-standardized YLDs stratified by gender, age and attributable to three risk factors in “B&R” countries from 1990 to 2021. Generally, different member states were at risk of stroke to varying degrees, with China, India and Indonesia being the three countries with the highest absolute numbers. The age-standardized incidence, mortality and DALYs of stroke were declining, with age-standardized YLDs rates differed among different gender and age groups in “B&R” member countries. Globally, there was a significant decline in the age-standardized YLDs rate for behavioral risks, environmental/occupational risks and metabolic risks. Therefore, in the context of a community with a shared future for mankind, strengthening health cooperation and the sharing of resources among member states will help jointly address the risks and challenges posed by stroke.

## Supplementary Material

Supplementary Material
