# Supplementary material for: Differences in the burden and temporal trend of stroke in the Belt and Road Initiative countries, 1990-2021
Source: Open Med (Wars). 2026 Sep 24;21(1):20261532. doi: 10.1515/med-2026-1532 (PMC13604947; doi:10.1515/med-2026-1532)
Supplement: Supplementary file 1 [file j_med-2026-1532_suppl_001.docx]

| Table S1 The age-standardized rates of incidence, mortality, YLDs and DALYs due to stroke in member countries of the "Belt and Road" initiative in 1990 and 2021 | | | | | | | | | |
| --- | --- | --- | --- | --- | --- | --- | --- | --- | --- |
|  |  | 1990(per 100,000 people) | | |  |  | 2021(per 100,000 people) | | |
|  | Incidence | Mortality | YLDs | DALYs |  | Incidence | Mortality | YLDs | DALYs |
| Global | 180.97 | 144.31 | 192.95 | 3078.95 |  | 141.55 | 87.45 | 178.68 | 1886.20 |
| **SDI regional** |  |  |  |  |  |  |  |  |  |
| High | 143.79 | 80.28 | 188.15 | 1606.41 |  | 90.20 | 32.57 | 163.31 | 730.56 |
| High-middle | 217.69 | 189.34 | 219.31 | 3722.31 |  | 162.00 | 100.45 | 205.64 | 1985.61 |
| middle | 192.21 | 178.61 | 191.99 | 3792.42 |  | 164.97 | 112.20 | 190.91 | 2302.80 |
| Low-middle | 170.88 | 139.29 | 151.96 | 3182.21 |  | 142.26 | 106.45 | 141.73 | 2340.82 |
| Low | 188.57 | 152.30 | 168.71 | 3492.28 |  | 147.41 | 111.87 | 147.56 | 2463.45 |
| **East Asia** |  |  |  |  |  |  |  |  |  |
| China | 226.94 | 242.18 | 228.84 | 4834.79 |  | 204.75 | 138.03 | 252.67 | 2648.02 |
| **Central Asia** |  |  |  |  |  |  |  |  |  |
| Armenia | 187.93 | 118.69 | 217.72 | 2396.68 |  | 111.41 | 65.28 | 182.09 | 1310.08 |
| Azerbaijan | 185.72 | 127.22 | 203.62 | 2770.92 |  | 197.07 | 101.29 | 190.70 | 2011.53 |
| Georgia | 254.95 | 192.08 | 251.82 | 3976.25 |  | 212.59 | 158.68 | 234.63 | 3054.07 |
| Kazakhstan | 278.53 | 181.21 | 340.16 | 3841.12 |  | 227.32 | 163.05 | 272.80 | 3099.20 |
| Kyrgyzstan | 258.36 | 199.21 | 277.83 | 4315.23 |  | 159.74 | 93.11 | 189.68 | 2190.02 |
| Mongolia | 238.72 | 214.85 | 240.01 | 4848.18 |  | 240.01 | 134.05 | 240.04 | 3036.34 |
| Tajikistan | 200.00 | 160.69 | 212.99 | 3524.00 |  | 223.72 | 130.12 | 195.69 | 2614.47 |
| Turkmenistan | 212.81 | 132.02 | 243.31 | 3169.86 |  | 225.33 | 160.54 | 288.21 | 3848.67 |
| Uzbekistan | 226.52 | 133.11 | 257.06 | 3198.96 |  | 248.95 | 105.42 | 248.11 | 2302.15 |
| **South Asia** |  |  |  |  |  |  |  |  |  |
| Bangladesh | 197.77 | 203.38 | 157.93 | 4576.14 |  | 167.28 | 149.02 | 143.92 | 2937.78 |
| Bhutan | 140.98 | 105.03 | 127.86 | 2372.85 |  | 110.01 | 71.19 | 115.84 | 1476.94 |
| India | 141.19 | 93.28 | 112.12 | 2200.85 |  | 107.37 | 72.00 | 104.07 | 1625.72 |
| Nepal | 142.97 | 126.92 | 119.26 | 2855.83 |  | 117.26 | 86.36 | 103.90 | 1801.12 |
| Pakistan | 179.97 | 106.60 | 165.00 | 2397.12 |  | 152.76 | 97.96 | 155.45 | 2194.44 |
| **Southeast Asia** |  |  |  |  |  |  |  |  |  |
| Cambodia | 229.64 | 237.65 | 215.99 | 5286.32 |  | 204.25 | 185.20 | 203.07 | 3709.43 |
| Indonesia | 243.48 | 206.24 | 312.03 | 4869.31 |  | 243.35 | 217.69 | 291.21 | 4587.07 |
| Laos | 255.10 | 297.32 | 260.93 | 7166.41 |  | 204.93 | 183.75 | 234.08 | 4061.51 |
| Malaysia | 231.52 | 140.16 | 282.24 | 3321.84 |  | 163.20 | 94.32 | 257.72 | 2122.31 |
| Maldives | 249.72 | 226.82 | 320.27 | 5353.13 |  | 131.11 | 80.10 | 192.18 | 1601.68 |
| Burma | 251.43 | 289.93 | 260.14 | 7027.87 |  | 187.75 | 181.09 | 221.15 | 4036.40 |
| Philippines | 124.41 | 133.63 | 182.33 | 2881.35 |  | 159.12 | 114.80 | 219.37 | 2761.76 |
| Sri Lanka | 166.86 | 176.86 | 229.74 | 3270.68 |  | 125.36 | 110.73 | 195.82 | 1992.14 |
| Thailand | 177.14 | 121.59 | 248.65 | 2751.64 |  | 114.92 | 65.59 | 204.36 | 1635.97 |
| Vietnam | 224.14 | 229.31 | 233.37 | 4783.36 |  | 203.36 | 196.56 | 236.32 | 3952.46 |
| **High-income Asia pacific** |  |  |  |  |  |  |  |  |  |
| Brunei | 264.87 | 145.39 | 376.15 | 3211.74 |  | 144.71 | 71.07 | 225.22 | 1508.96 |
| Singapore | 213.45 | 90.58 | 327.01 | 2103.65 |  | 78.80 | 14.23 | 167.79 | 428.57 |
| **North Africa and Middle East** |  |  |  |  |  |  |  |  |  |
| Afghanistan | 229.28 | 255.82 | 176.20 | 5857.28 |  | 179.82 | 183.88 | 147.64 | 3982.47 |
| Bahrain | 122.56 | 160.01 | 128.43 | 2941.16 |  | 79.02 | 81.47 | 100.33 | 1384.17 |
| Egypt | 157.97 | 272.56 | 145.10 | 5450.23 |  | 163.41 | 174.15 | 158.32 | 3282.10 |
| Iran | 147.72 | 124.68 | 151.70 | 2470.21 |  | 100.81 | 63.89 | 129.61 | 1229.18 |
| Iraq | 203.97 | 198.63 | 189.80 | 4564.21 |  | 187.48 | 169.98 | 175.07 | 3324.59 |
| Jordan | 208.84 | 149.56 | 198.43 | 3127.35 |  | 158.53 | 69.27 | 191.33 | 1383.13 |
| Kuwait | 111.33 | 50.61 | 162.66 | 1196.52 |  | 101.04 | 34.04 | 149.69 | 754.69 |
| Lebanon | 154.10 | 141.91 | 153.88 | 2946.03 |  | 116.95 | 44.50 | 149.81 | 917.05 |
| Oman | 172.07 | 126.39 | 166.45 | 2842.38 |  | 149.57 | 74.15 | 159.10 | 1470.77 |
| Palestine | 182.49 | 197.38 | 137.93 | 3660.72 |  | 157.98 | 114.01 | 119.89 | 1991.71 |
| Qatar | 174.75 | 158.08 | 220.48 | 2937.97 |  | 110.60 | 54.76 | 137.25 | 1004.21 |
| Saudi Arabia | 166.31 | 161.70 | 133.50 | 3342.44 |  | 130.68 | 99.48 | 129.95 | 2023.34 |
| Syria | 181.90 | 150.17 | 188.98 | 3546.26 |  | 135.16 | 106.33 | 143.46 | 2124.44 |
| Turkey | 177.83 | 144.70 | 203.71 | 3116.20 |  | 110.43 | 72.10 | 142.59 | 1343.01 |
| United Arab Emirates | 241.11 | 136.25 | 229.42 | 2921.93 |  | 173.46 | 88.79 | 190.17 | 1648.81 |
| Yemen | 186.96 | 220.61 | 144.86 | 4871.77 |  | 157.24 | 165.08 | 132.37 | 3331.12 |
| **Central Europe** |  |  |  |  |  |  |  |  |  |
| Albania | 180.63 | 201.14 | 174.69 | 3539.31 |  | 155.33 | 150.73 | 148.18 | 2316.18 |
| Bosnia and Herzegovina | 206.62 | 163.90 | 241.79 | 3188.99 |  | 171.29 | 110.13 | 226.75 | 2022.81 |
| Bulgaria | 285.50 | 305.22 | 254.49 | 5517.01 |  | 225.90 | 192.08 | 224.98 | 3430.21 |
| Croatia | 214.86 | 195.26 | 200.61 | 3543.25 |  | 122.28 | 65.95 | 160.68 | 1174.27 |
| Czechia | 228.68 | 195.09 | 228.34 | 3494.34 |  | 110.12 | 40.61 | 171.26 | 812.22 |
| Hungary | 242.98 | 188.84 | 275.65 | 3689.84 |  | 117.24 | 55.27 | 169.23 | 1143.42 |
| Montenegro | 221.87 | 184.19 | 179.77 | 3472.30 |  | 207.16 | 225.77 | 160.15 | 3517.19 |
| North Macedonia | 307.76 | 282.49 | 280.26 | 5343.48 |  | 281.41 | 277.48 | 226.84 | 4267.25 |
| Poland | 158.58 | 168.02 | 161.96 | 3095.87 |  | 102.95 | 58.04 | 137.38 | 1118.58 |
| Romania | 255.83 | 235.52 | 237.11 | 4274.74 |  | 170.50 | 128.32 | 197.80 | 2361.16 |
| Serbia | 265.46 | 318.01 | 245.72 | 5409.55 |  | 194.15 | 160.19 | 181.66 | 2695.72 |
| Slovakia | 219.57 | 144.39 | 299.21 | 2973.06 |  | 137.79 | 65.95 | 218.99 | 1345.62 |
| Slovenia | 174.20 | 109.58 | 162.05 | 2094.70 |  | 76.52 | 33.39 | 113.89 | 591.31 |
| **Eastern Europe** |  |  |  |  |  |  |  |  |  |
| Belarus | 230.54 | 133.41 | 239.58 | 2843.90 |  | 174.78 | 97.81 | 215.37 | 2037.39 |
| Estonia | 215.28 | 170.40 | 199.75 | 3227.93 |  | 89.05 | 35.77 | 140.94 | 751.11 |
| Latvia | 265.99 | 199.90 | 222.23 | 3722.97 |  | 164.09 | 106.11 | 198.42 | 1912.72 |
| Lithuania | 245.22 | 97.74 | 196.85 | 2081.67 |  | 176.13 | 68.20 | 184.76 | 1335.27 |
| Republic of Moldova | 218.47 | 177.78 | 188.65 | 3550.99 |  | 148.84 | 85.19 | 171.80 | 1888.89 |
| Russia | 250.60 | 231.57 | 219.32 | 4218.57 |  | 183.00 | 129.79 | 198.53 | 2519.58 |
| Ukraine | 261.26 | 189.40 | 252.83 | 3503.70 |  | 183.72 | 104.21 | 217.07 | 2152.97 |
| **Western Europe** |  |  |  |  |  |  |  |  |  |
| Cyprus | 130.59 | 177.27 | 118.40 | 2497.44 |  | 67.46 | 47.75 | 71.40 | 684.94 |
| Greece | 180.87 | 155.40 | 157.14 | 2464.89 |  | 103.19 | 51.52 | 120.91 | 909.47 |
| Israel | 132.37 | 61.71 | 155.71 | 1202.75 |  | 65.13 | 20.26 | 109.45 | 426.46 |

| Table S2 The average annual percentage change (AAPC) of age-standardized incidence, mortality, YLDs rate and DALYs rate due to stroke for 1990-2021 for “the Belt & Road” countries | | | | | | | | |
| --- | --- | --- | --- | --- | --- | --- | --- | --- |
|  | **Age-standardized incidence** | | **Age-standardized mortality** | | **Age-standardized YLDs rate** | | **Age-standardized DALYs rate** | |
| **Global/Regions/Countries** | **AAPC (95%CI, %)** | ***P*** | **AAPC (95%CI, %)** | ***P*** | **AAPC (95%CI, %)** | ***P*** | **AAPC (95%CI, %)** | ***P*** |
| Global | -0.81(-0.86, -0.75) | ＜0.001 | -1.60 (-1.79, -1.41) | ＜0.001 | -0.26 (-0.27, -0.24) | ＜0.001 | -1.57 (-1.69, -1.45) | ＜0.001 |
| **SDI Regions** |  |  |  |  |  |  |  |  |
| High SDI | -1.49 (-1.57, -1.41) | ＜0.001 | -2.90 (-3.07, -2.72) | ＜0.001 | -0.46 (-0.48, -0.45) | ＜0.001 | -2.53 (-2.67, -2.39) | ＜0.001 |
| High-middle SDI | -0.97 (-1.04, -0.9) | ＜0.001 | -2.02 (-2.31, -1.74) | ＜0.001 | -0.22 (-0.23, -0.21) | ＜0.001 | -1.99 (-2.25, -1.73) | ＜0.001 |
| Middle SDI | -0.52 (-0.58, -0.46) | ＜0.001 | -1.50 (-1.68, -1.32) | ＜0.001 | -0.03 (-0.04, -0.01) | ＜0.001 | -1.61 (-1.75, -1.47) | ＜0.001 |
| Low-middle SDI | -0.60 (-0.63, -0.58) | ＜0.001 | -0.84 (-0.92, -0.76) | ＜0.001 | -0.22 (-0.23, -0.22) | ＜0.001 | -0.95 (-1.10, -0.81) | ＜0.001 |
| Low SDI | -0.80 (-0.83, -0.78) | ＜0.001 | -0.97 (-1.09, -0.85) | ＜0.001 | -0.43 (-0.44, -0.42) | ＜0.001 | -1.10 (-1.23, -0.98) | ＜0.001 |
| **East Asia** |  |  |  |  |  |  |  |  |
| China | -0.37 (-0.44, -0.29) | ＜0.001 | -1.79 (-2.01, -1.57) | ＜0.001 | 0.30 (0.26, 0.33) | ＜0.001 | -1.93 (-2.09, -1.77) | ＜0.001 |
| **Central Asia** |  |  |  |  |  |  |  |  |
| Armenia | -1.68 (-1.76, -1.6) | ＜0.001 | -1.84 (-2.64, -1.04) | ＜0.001 | -0.57 (-0.60, -0.54) | ＜0.001 | -1.84 (-2.38, -1.3) | ＜0.001 |
| Azerbaijan | 0.19 (0.12, 0.25) | ＜0.001 | -0.71 (-1.02, -0.41) | ＜0.001 | -0.21 (-0.24, -0.18) | ＜0.001 | -1.02 (-1.28, -0.77) | ＜0.001 |
| Georgia | -0.59 (-0.63, -0.56) | ＜0.001 | -0.47 (-1.75, 0.82) | 0.470 | -0.23 (-0.24, -0.22) | ＜0.001 | -0.84 (-1.60, -0.08) | 0.031 |
| Kazakhstan | -0.67 (-0.75, -0.59) | ＜0.001 | -0.22 (-1.2, 0.77) | 0.662 | -0.70 (-0.75, -0.65) | ＜0.001 | -0.61 (-1.32, 0.10) | 0.090 |
| Kyrgyzstan | -1.57 (-1.61, -1.53) | ＜0.001 | -2.41 (-2.85, -1.98) | ＜0.001 | -1.22 (-1.25, -1.18) | ＜0.001 | -2.14 (-2.49, -1.79) | ＜0.001 |
| Mongolia | -0.01 (-0.09, 0.07) | 0.818 | -1.73 (-1.96, -1.51) | ＜0.001 | 0.00 (-0.05, 0.05) | 0.907 | -1.76 (-2.06, -1.46) | ＜0.001 |
| Tajikistan | 0.36 (0.32, 0.41) | ＜0.001 | -0.70 (-1.12, -0.28) | 0.001 | -0.28 (-0.32, -0.25) | ＜0.001 | -1.00 (-1.27, -0.73) | ＜0.001 |
| Turkmenistan | 0.20 (0.11, 0.3) | ＜0.001 | 0.79 (-0.10, 1.69) | 0.083 | 0.55 (0.52, 0.59) | ＜0.001 | 0.78 (-0.02, 1.58) | 0.057 |
| Uzbekistan | 0.29 (0.21, 0.38) | ＜0.001 | -0.68 (-1.31, -0.04) | 0.036 | -0.11 (-0.13, -0.1) | ＜0.001 | -1.08 (-1.52, -0.64) | ＜0.001 |
| **South Asia** |  |  |  |  |  |  |  |  |
| Bangladesh | -0.57 (-0.65, -0.49) | ＜0.001 | -0.80 (-1.44, -0.15) | 0.016 | -0.30 (-0.32, -0.28) | ＜0.001 | -1.32 (-1.69, -0.96) | ＜0.001 |
| Bhutan | -0.81 (-0.84, -0.78) | ＜0.001 | -1.23 (-1.31, -1.14) | ＜0.001 | -0.32 (-0.34, -0.3) | ＜0.001 | -1.51 (-1.6, -1.42) | ＜0.001 |
| India | -0.89 (-0.94, -0.84) | ＜0.001 | -0.81 (-1.3, -0.31) | 0.002 | -0.24 (-0.25, -0.23) | ＜0.001 | -0.92 (-1.21, -0.63) | ＜0.001 |
| Nepal | -0.64 (-0.67, -0.6) | ＜0.001 | -1.22 (-1.3, -1.15) | ＜0.001 | -0.44 (-0.46, -0.42) | ＜0.001 | -1.47 (-1.57, -1.37) | ＜0.001 |
| Pakistan | -0.54 (-0.58, -0.49) | ＜0.001 | -0.26 (-0.36, -0.16) | ＜0.001 | -0.19 (-0.21, -0.17) | ＜0.001 | -0.28 (-0.39, -0.17) | ＜0.001 |
| **Southeast Asia** |  |  |  |  |  |  |  |  |
| Cambodia | -0.39 (-0.4, -0.38) | ＜0.001 | -0.80 (-0.85, -0.75) | ＜0.001 | -0.20 (-0.24, -0.17) | ＜0.001 | -1.14(-1.19, -1.09) | ＜0.001 |
| Indonesia | 0 (-0.05, 0.04) | 0.927 | 0.17 (0.11, 0.23) | ＜0.001 | -0.22 (-0.25, -0.19) | ＜0.001 | -0.20(-0.26, -0.14) | ＜0.001 |
| Lao People's Democratic Republic | -0.72 (-0.77, -0.67) | ＜0.001 | -1.54 (-1.60, -1.48) | ＜0.001 | -0.35 (-0.37, -0.33) | ＜0.001 | -1.82 (-1.85, -1.79) | ＜0.001 |
| Malaysia | -1.14 (-1.16, -1.11) | ＜0.001 | -1.18 (-1.31, -1.05) | ＜0.001 | -0.29 (-0.31, -0.26) | ＜0.001 | -1.37 (-1.59, -1.15) | ＜0.001 |
| Maldives | -2.07(-2.14, -2.00) | ＜0.001 | -3.48 (-3.67, -3.29) | ＜0.001 | -1.63 (-1.69, -1.57) | ＜0.001 | -3.97 (-4.16, -3.78) | ＜0.001 |
| Myanmar | -0.97 (-1.01, -0.93) | ＜0.001 | -1.51 (-1.57, -1.45) | ＜0.001 | -0.52 (-0.55, -0.49) | ＜0.001 | -1.78 (-1.84, -1.71) | ＜0.001 |
| Philippines | 0.80 (0.73, 0.87) | ＜0.001 | -0.48 (-0.89, -0.07) | 0.022 | 0.61 (0.58, 0.63) | ＜0.001 | -0.12 (-0.47, 0.24) | 0.521 |
| Sri Lanka | -0.95 (-0.98, -0.92) | ＜0.001 | -1.56 (-2.24, -0.88) | ＜0.001 | -0.51 (-0.56, -0.47) | ＜0.001 | -1.56 (-2.31, -0.8) | ＜0.001 |
| Thailand | -1.41 (-1.46, -1.36) | ＜0.001 | -1.97 (-2.36, -1.59) | ＜0.001 | -0.62 (-0.66, -0.59) | ＜0.001 | -1.71 (-2.05, -1.37) | ＜0.001 |
| Viet Nam | -0.34 (-0.48, -0.2) | ＜0.001 | -0.49 (-0.53, -0.45) | ＜0.001 | 0.04 (0.01, 0.06) | 0.005 | -0.61 (-0.64, -0.58) | ＜0.001 |
| **High-income Asia pacific** | |  |  |  |  |  |  |  |
| Brunei Darussalam | -1.94 (-1.99, -1.88) | ＜0.001 | -2.41 (-2.76, -2.07) | ＜0.001 | -1.64 (-1.68, -1.6) | ＜0.001 | -2.47 (-2.80, -2.15) | ＜0.001 |
| Singapore | -3.21 (-3.03, -3.12) | ＜0.001 | -5.81 (-6.6, -5.01) | ＜0.001 | -2.13 (-2.20, -2.05) | ＜0.001 | -4.97 (-5.41, -4.53) | ＜0.001 |
| **North Africa and Middle East** | |  |  |  |  |  |  |  |
| Afghanistan | -0.81 (-0.87, -0.75) | ＜0.001 | -1.06 (-1.12, -1.00) | ＜0.001 | -0.57 (-0.6, -0.54) | ＜0.001 | -1.23 (-1.30, -1.17) | ＜0.001 |
| Bahrain | -1.43 (-1.49, -1.37) | ＜0.001 | -2.23 (-2.89, -1.57) | ＜0.001 | -0.78 (-0.83, -0.74) | ＜0.001 | -2.50 (-3.07, -1.91) | ＜0.001 |
| Egypt | 0.09 (0.05, 0.14) | ＜0.001 | -1.44 (-1.96, -0.92) | ＜0.001 | 0.28 (0.26, 0.29) | ＜0.001 | -1.56 (-2.15, -0.96) | ＜0.001 |
| Iran (Islamic Republic of) | -1.23 (-1.31, -1.16) | ＜0.001 | -2.15 (-2.28, -2.03) | ＜0.001 | -0.50 (-0.54, -0.46) | ＜0.001 | -2.24 (-2.35, -2.14) | ＜0.001 |
| Iraq | -0.28 (-0.32, -0.24) | ＜0.001 | -0.43 (-0.65, -0.21) | ＜0.001 | -0.26 (-0.28, -0.23) | ＜0.001 | -1.00 (-1.16, -0.83) | ＜0.001 |
| Jordan | -0.90 (-0.98, -0.82) | ＜0.001 | -2.60 (-3.16, -2.04) | ＜0.001 | -0.09 (-0.14, -0.03) | 0.001 | -2.56 (-3.08, -2.03) | ＜0.001 |
| Kuwait | -0.32 (-0.58, -0.06) | 0.014 | -1.16 (-3.08, 0.79) | 0.242 | -0.24 (-0.3, -0.18) | ＜0.001 | -1.39 (-2.86, 0.11) | 0.070 |
| Lebanon | -0.90 (-0.91, -0.89) | ＜0.001 | -3.67 (-3.94, -3.41) | ＜0.001 | -0.08 (-0.11, -0.06) | ＜0.001 | -3.70 (-4.01, -3.39) | ＜0.001 |
| Oman | -0.47 (-0.51, -0.44) | ＜0.001 | -1.66 (-2.74, -0.56) | 0.003 | -0.14 (-0.17, -0.12) | ＜0.001 | -2.07 (-2.52, -1.62) | ＜0.001 |
| Palestine | -0.50 (-0.55, -0.44) | ＜0.001 | -1.79 (-1.91, -1.66) | ＜0.001 | -0.46 (-0.48, -0.43) | ＜0.001 | -1.98 (-2.22, -1.73) | ＜0.001 |
| Qatar | -1.49 (-1.66, -1.33) | ＜0.001 | -3.38 (-4.74, -2.00) | ＜0.001 | -1.44 (-1.62, -1.27) | ＜0.001 | -3.41 (-4.42, -2.39) | ＜0.001 |
| Saudi Arabia | -0.82 (-0.91, -0.74) | ＜0.001 | -1.58 (-1.77, -1.4) | ＜0.001 | -0.09 (-0.13, -0.05) | ＜0.001 | -1.64 (-1.80, -1.49) | ＜0.001 |
| Syrian Arab Republic | -0.95 (-0.99, -0.91) | ＜0.001 | -1.06 (-1.30, -0.83) | ＜0.001 | -0.89 (-0.93, -0.85) | ＜0.001 | -1.62 (-1.90, -1.35) | ＜0.001 |
| Türkiye | -1.55 (-1.64, -1.47) | ＜0.001 | -2.16 (-2.48, -1.83) | ＜0.001 | -1.14 (-1.19, -1.09) | ＜0.001 | -2.69 (-2.91, -2.46) | ＜0.001 |
| United Arab Emirates | -1.06 (-1.12, -0.99) | ＜0.001 | -1.43 (-3.29, 0.47) | 0.139 | -0.60 (-0.63, -0.58) | ＜0.001 | -1.87 (-3.39, -0.33) | 0.017 |
| Yemen | -0.56 (-0.57, -0.55) | ＜0.001 | -0.92 (-0.99, -0.84) | ＜0.001 | -0.28 (-0.30, -0.26) | ＜0.001 | -1.20 (-1.34, -1.06) | ＜0.001 |
| **Central Europe** |  |  |  |  |  |  |  |  |
| Albania | -0.48 (-0.51, -0.46) | ＜0.001 | -1.02 (-1.61, -0.43) | 0.001 | -0.52 (-0.55, -0.48) | ＜0.001 | -1.49 (-2.05, -0.93) | ＜0.001 |
| Bosnia and Herzegovina | -0.60 (-0.72, -0.49) | ＜0.001 | -1.24 (-1.49, -1.00) | ＜0.001 | -0.2 (-0.23, -0.18) | ＜0.001 | -1.43 (-1.76, -1.10) | ＜0.001 |
| Bulgaria | -0.77 (-0.82, -0.73) | ＜0.001 | -1.48 (-2.15, -0.81) | ＜0.001 | -0.4 (-0.41, -0.39) | ＜0.001 | -1.49 (-1.77, -1.20) | ＜0.001 |
| Croatia | -1.80 (-1.84, -1.76) | ＜0.001 | -3.54 (-3.77, -3.32) | ＜0.001 | -0.71 (-0.75, -0.66) | ＜0.001 | -3.46 (-3.91, -3.01) | ＜0.001 |
| Czechia | -2.37 (-2.42, -2.32) | ＜0.001 | -4.96 (-5.37, -4.55) | ＜0.001 | -0.89 (-0.92, -0.86) | ＜0.001 | -4.63 (-4.91, -4.34) | ＜0.001 |
| Hungary | -2.36 (-2.40, -2.31) | ＜0.001 | -3.91 (-4.48, -3.34) | ＜0.001 | -1.56 (-1.63, -1.48) | ＜0.001 | -3.65 (-4.15, -3.15) | ＜0.001 |
| Montenegro | -0.23 (-0.26, -0.2) | ＜0.001 | 0.78 (-0.03, 1.59) | 0.059 | -0.35 (-0.42, -0.27) | ＜0.001 | 0.17 (-0.12, 0.46) | 0.248 |
| North Macedonia | -0.30 (-0.34, -0.26) | ＜0.001 | -0.07 (-0.52, 0.39) | 0.763 | -0.68 (-0.70, -0.66) | ＜0.001 | -0.69 (-1.27, -0.11) | 0.021 |
| Poland | -1.40 (-1.46, -1.34) | ＜0.001 | -3.43 (-3.9, -2.97) | ＜0.001 | -0.53 (-0.55, -0.51) | ＜0.001 | -3.36 (-3.75, -2.97) | ＜0.001 |
| Romania | -1.32 (-1.37, -1.27) | ＜0.001 | -2.01 (-2.53, -1.49) | ＜0.001 | -0.57 (-0.61, -0.53) | ＜0.001 | -1.96 (-2.42, -1.5) | ＜0.001 |
| Serbia | -1.02 (-1.06, -0.97) | ＜0.001 | -2.13 (-2.41, -1.84) | ＜0.001 | -0.97 (-1.01, -0.94) | ＜0.001 | -2.19 (-2.39, -1.99) | ＜0.001 |
| Slovakia | -1.51 (-1.55, -1.47) | ＜0.001 | -2.52(-3.00, -2.04) | ＜0.001 | -0.99 (-1.11, -0.88) | ＜0.001 | -2.50 (-2.71, -2.29) | ＜0.001 |
| Slovenia | -2.63 (-2.69, -2.57) | ＜0.001 | -3.80(-4.66, -2.94) | ＜0.001 | -1.11 (-1.16, -1.06) | ＜0.001 | -4.02 (-4.69, -3.34) | ＜0.001 |
| **Eastern Europe** |  |  |  |  |  |  |  |  |
| Belarus | -0.90 (-1.01, -0.78) | ＜0.001 | -0.99 (-1.46, -0.51) | ＜0.001 | -0.33 (-0.38, -0.27) | ＜0.001 | -1.04 (-1.51, -0.57) | ＜0.001 |
| Estonia | -2.84 (-2.90, -2.78) | ＜0.001 | -4.92 (-5.75, -4.08) | ＜0.001 | -1.09 (-1.14, -1.05) | ＜0.001 | -4.58 (-5.53, -3.62) | ＜0.001 |
| Latvia | -1.56 (-1.67, -1.46) | ＜0.001 | -1.83 (-3.19, -0.44) | 0.010 | -0.33 (-0.42, -0.23) | ＜0.001 | -1.97 (-3.17, -0.75) | 0.002 |
| Lithuania | -1.09 (-1.14, -1.04) | ＜0.001 | -1.12 (-1.60, -0.64) | ＜0.001 | -0.08 (-0.37, 0.21) | 0.570 | -1.29 (-1.79, -0.78) | ＜0.001 |
| Republic of Moldova | -1.23 (-1.38, -1.08) | ＜0.001 | -2.40 (-3.17, -1.62) | ＜0.001 | -0.30 (-0.34, -0.26) | ＜0.001 | -2.07 (-3.06, -1.07) | ＜0.001 |
| Russian Federation | -1.02 (-1.09, -0.95) | ＜0.001 | -1.76 (-2.57, -0.93) | ＜0.001 | -0.34 (-0.36, -0.31) | ＜0.001 | -1.56 (-2.45, -0.66) | 0.001 |
| Ukraine | -1.12 (-1.17, -1.08) | ＜0.001 | -1.95 (-2.62, -1.28) | ＜0.001 | -0.47 (-0.52, -0.42) | ＜0.001 | -1.59 (-2.20, -0.98) | ＜0.001 |
| **Western Europe** |  |  |  |  |  |  |  |  |
| Cyprus | -2.10 (-2.31, -1.88) | ＜0.001 | -4.07 (-4.89, -3.24) | ＜0.001 | -1.61 (-1.69, -1.54) | ＜0.001 | -4.07 (-4.65, -3.48) | ＜0.001 |
| Greece | -1.80 (-1.86, -1.74) | ＜0.001 | -3.63 (-3.90, -3.37) | ＜0.001 | -0.84 (-0.88, -0.79) | ＜0.001 | -3.26 (-3.53, -2.98) | ＜0.001 |
| Israel | -2.30 (-2.61, -1.98) | ＜0.001 | -3.56 (-4.50, -2.61) | ＜0.001 | -1.05 (-1.13, -0.97) | ＜0.001 | -3.27 (-3.91, -2.63) | ＜0.001 |

AAPC: average annual percentage change; CI: confidence interval

| Table S3 The average annual percentage change (AAPC) of age-standardized rates for YLDs due to stroke, stratified by gender for 1990-2021 for “the Belt & Road” countries | | | | | |
| --- | --- | --- | --- | --- | --- |
| **Global/Regions/Countries** | **Male** | |  | **Female** | |
|  | **AAPC (95%CI, %)** | ***P*** |  | **AAPC (95%CI, %)** | ***P*** |
| Global | -0.16 (-0.17 to -0.14) | ＜0.001 |  | -0.36 (-0.38 to -0.34) | ＜0.001 |
| **SDI Regions** |  |  |  |  |  |
| High SDI | -0.48 (-0.50 to -0.46) | ＜0.001 |  | -0.48 (-0.51 to -0.45) | ＜0.001 |
| High-middle SDI | -0.14 (-0.16 to -0.12) | ＜0.001 |  | -0.32 (-0.34 to -0.31) | ＜0.001 |
| Middle SDI | 0.16 (0.15 to 0.18) | ＜0.001 |  | -0.21 (-0.23 to -0.2) | ＜0.001 |
| Low-middle SDI | -0.11 (-0.11 to -0.1) | ＜0.001 |  | -0.33 (-0.35 to -0.31) | ＜0.001 |
| Low SDI | -0.41 (-0.44 to -0.39) | ＜0.001 |  | -0.45 (-0.46 to -0.44) | ＜0.001 |
| **East Asia** |  |  |  |  |  |
| China | 0.41 (0.38 to 0.45) | ＜0.001 |  | 0.12 (0.06 to 0.18) | ＜0.001 |
| **Central Asia** |  |  |  |  |  |
| Armenia | -0.21 (-0.26 to -0.17) | ＜0.001 |  | -0.81 (-0.84 to -0.78) | ＜0.001 |
| Azerbaijan | -0.17 (-0.19 to -0.14) | ＜0.001 |  | -0.20 (-0.23 to -0.16) | ＜0.001 |
| Georgia | 0 (-0.02 to 0.02) | 0.912 |  | -0.37 (-0.38 to -0.35) | ＜0.001 |
| Kazakhstan | -0.79 (-0.86 to -0.72) | ＜0.001 |  | -0.63 (-0.67 to -0.58) | ＜0.001 |
| Kyrgyzstan | -1.12 (-1.16 to -1.08) | ＜0.001 |  | -1.29 (-1.32 to -1.26) | ＜0.001 |
| Mongolia | 0.37 (0.32 to 0.43) | ＜0.001 |  | -0.27 (-0.32 to -0.23) | ＜0.001 |
| Tajikistan | -0.34 (-0.36 to -0.32) | ＜0.001 |  | -0.23 (-0.31 to -0.15) | ＜0.001 |
| Turkmenistan | 0.60 (0.58 to 0.61) | ＜0.001 |  | 0.54 (0.49 to 0.58) | ＜0.001 |
| Uzbekistan | -0.11 (-0.15 to -0.08) | ＜0.001 |  | -0.13 (-0.15 to -0.11) | ＜0.001 |
| **South Asia** |  |  |  |  |  |
| Bangladesh | -0.34 (-0.36 to -0.32) | ＜0.001 |  | -0.21 (-0.24 to -0.19) | ＜0.001 |
| Bhutan | -0.30 (-0.32 to -0.27) | ＜0.001 |  | -0.38 (-0.41 to -0.35) | ＜0.001 |
| India | -0.11 (-0.12 to -0.1) | ＜0.001 |  | -0.33 (-0.35 to -0.32) | ＜0.001 |
| Nepal | -0.38 (-0.39 to -0.36) | ＜0.001 |  | -0.51 (-0.54 to -0.48) | ＜0.001 |
| Pakistan | -0.22 (-0.23 to -0.21) | ＜0.001 |  | -0.16 (-0.17 to -0.14) | ＜0.001 |
| **Southeast Asia** |  |  |  |  |  |
| Cambodia | -0.21 (-0.23 to -0.19) | ＜0.001 |  | -0.18 (-0.21 to -0.15) | ＜0.001 |
| Indonesia | -0.08 (-0.1 to -0.06) | ＜0.001 |  | -0.32 (-0.33 to -0.31) | ＜0.001 |
| Lao People's Democratic Republic | -0.32 (-0.36 to -0.28) | ＜0.001 |  | -0.38 (-0.40 to -0.36) | ＜0.001 |
| Malaysia | -0.32 (-0.34 to -0.29) | ＜0.001 |  | -0.26 (-0.27 to -0.25) | ＜0.001 |
| Maldives | -1.48 (-1.54 to -1.41) | ＜0.001 |  | -1.75 (-1.83 to -1.67) | ＜0.001 |
| Myanmar | -0.34 (-0.37 to -0.31) | ＜0.001 |  | -0.65 (-0.68 to -0.63) | ＜0.001 |
| Philippines | 0.64 (0.61 to 0.68) | ＜0.001 |  | 0.57 (0.55 to 0.59) | ＜0.001 |
| Sri Lanka | -0.57 (-0.60 to -0.53) | ＜0.001 |  | -0.46 (-0.48 to -0.44) | ＜0.001 |
| Thailand | -0.48 (-0.52 to -0.45) | ＜0.001 |  | -0.74 (-0.78 to -0.71) | ＜0.001 |
| Viet Nam | 0.28 (0.25 to 0.31) | ＜0.001 |  | -0.19 (-0.22 to -0.17) | ＜0.001 |
| **High-income Asia pacific** |  |  |  |  |  |
| Brunei Darussalam | -1.58 (-1.64 to -1.52) | ＜0.001 |  | -1.63 (-1.66 to -1.59) | ＜0.001 |
| Singapore | -2.28 (-2.37 to -2.18) | ＜0.001 |  | -2.02 (-2.08 to -1.96) | ＜0.001 |
| **North Africa and Middle East** |  |  |  |  |  |
| Afghanistan | -0.33 (-0.36 to -0.31) | ＜0.001 |  | -0.69 (-0.72 to -0.66) | ＜0.001 |
| Bahrain | -0.69 (-0.73 to -0.65) | ＜0.001 |  | -0.84 (-0.86 to -0.81) | ＜0.001 |
| Egypt | 0.55 (0.50 to 0.59) | ＜0.001 |  | 0.02 (-0.01 to 0.04) | 0.122 |
| Iran (Islamic Republic of) | -0.21 (-0.23 to -0.19) | ＜0.001 |  | -0.80 (-0.84 to -0.77) | ＜0.001 |
| Iraq | -0.16 (-0.19 to -0.13) | ＜0.001 |  | -0.34 (-0.35 to -0.33) | ＜0.001 |
| Jordan | 0.20 (0.13 to 0.27) | ＜0.001 |  | -0.38 (-0.44 to -0.33) | ＜0.001 |
| Kuwait | -0.21 (-0.25 to -0.17) | ＜0.001 |  | -0.50 (-0.56 to -0.43) | ＜0.001 |
| Lebanon | -0.02 (-0.05 to 0.01) | 0.279 |  | -0.13 (-0.15 to -0.10) | ＜0.001 |
| Oman | -0.02 (-0.06 to 0.02) | 0.280 |  | -0.31 (-0.33 to -0.28) | ＜0.001 |
| Palestine | -0.45 (-0.50 to -0.40) | ＜0.001 |  | -0.42 (-0.43 to -0.4) | ＜0.001 |
| Qatar | -1.56 (-1.78 to -1.33) | ＜0.001 |  | -1.26 (-1.35 to -1.16) | ＜0.001 |
| Saudi Arabia | -0.06 (-0.12 to -0.01) | 0.027 |  | -0.18 (-0.23 to -0.13) | ＜0.001 |
| Syrian Arab Republic | -0.84 (-0.87 to -0.8) | ＜0.001 |  | -0.95(-1.00 to -0.91) | ＜0.001 |
| Türkiye | -1.31 (-1.39 to -1.23) | ＜0.001 |  | -1.00 (-1.04 to -0.95) | ＜0.001 |
| United Arab Emirates | -0.75 (-0.78 to -0.72) | ＜0.001 |  | -0.58 (-0.60 to -0.56) | ＜0.001 |
| Yemen | -0.32 (-0.33 to -0.31) | ＜0.001 |  | -0.28(-0.29 to -0.26) | ＜0.001 |
| **Central Europe** |  |  |  |  |  |
| Albania | -0.51 (-0.54 to -0.47) | ＜0.001 |  | -0.56 (-0.60 to -0.52) | ＜0.001 |
| Bosnia and Herzegovina | -0.14 (-0.19 to -0.09) | ＜0.001 |  | -0.25 (-0.30 to -0.21) | ＜0.001 |
| Bulgaria | -0.53 (-0.55 to -0.51) | ＜0.001 |  | -0.25 (-0.26 to -0.23) | ＜0.001 |
| Croatia | -0.66 (-0.72 to -0.6) | ＜0.001 |  | -0.81 (-0.85 to -0.76) | ＜0.001 |
| Czechia | -1.01 (-1.06 to -0.97) | ＜0.001 |  | -0.83 (-0.88 to -0.78) | ＜0.001 |
| Hungary | -1.72 (-1.81 to -1.63) | ＜0.001 |  | -1.39 (-1.43 to -1.34) | ＜0.001 |
| Montenegro | -0.51 (-0.61 to -0.4) | ＜0.001 |  | -0.22 (-0.25 to -0.18) | ＜0.001 |
| North Macedonia | -0.67 (-0.70 to -0.64) | ＜0.001 |  | -0.68 (-0.71 to -0.65) | ＜0.001 |
| Poland | -0.42 (-0.45 to -0.38) | ＜0.001 |  | -0.62 (-0.69 to -0.54) | ＜0.001 |
| Romania | -0.50 (-0.54 to -0.46) | ＜0.001 |  | -0.61 (-0.65 to -0.57) | ＜0.001 |
| Serbia | -0.89 (-0.93 to -0.86) | ＜0.001 |  | -1.04 (-1.08 to -1.00) | ＜0.001 |
| Slovakia | -1.02 (-1.22 to -0.82) | ＜0.001 |  | -0.85 (-0.92 to -0.78) | ＜0.001 |
| Slovenia | -1.16 (-1.21 to -1.11) | ＜0.001 |  | -1.14 (-1.18 to -1.11) | ＜0.001 |
| **Eastern Europe** |  |  |  |  |  |
| Belarus | -0.35 (-0.45 to -0.25) | ＜0.001 |  | -0.33 (-0.36 to -0.3) | ＜0.001 |
| Estonia | -1.31 (-1.38 to -1.23) | ＜0.001 |  | -0.85 (-0.88 to -0.81) | ＜0.001 |
| Latvia | 0.06 (-0.03 to 0.15) | 0.200 |  | -0.56 (-0.62 to -0.5) | ＜0.001 |
| Lithuania | 0.36 (0.18 to 0.54) | ＜0.001 |  | -0.43 (-0.86 to 0.01) | 0.054 |
| Republic of Moldova | -0.09 (-0.14 to -0.03) | 0.001 |  | -0.43 (-0.46 to -0.4) | ＜0.001 |
| Russian Federation | -0.21 (-0.23 to -0.19) | ＜0.001 |  | -0.42 (-0.46 to -0.37) | ＜0.001 |
| Ukraine | -0.55 (-0.64 to -0.46) | ＜0.001 |  | -0.49 (-0.52 to -0.46) | ＜0.001 |
| **Western Europe** |  |  |  |  |  |
| Cyprus | -1.67 (-1.73 to -1.62) | ＜0.001 |  | -1.60 (-1.7 to -1.51) | ＜0.001 |
| Greece | -0.74 (-0.79 to -0.7) | ＜0.001 |  | -0.94 (-0.98 to -0.9) | ＜0.001 |
| Israel | -1.19 (-1.29 to -1.1) | ＜0.001 |  | -0.88 (-0.96 to -0.8) | ＜0.001 |

AAPC: average annual percentage change; CI: confidence interval

| Table S4 The average annual percentage change (AAPC) of YLDs due to stroke, stratified by age for 1990-2021 for “the Belt & Road” countries | | | | | | | | |
| --- | --- | --- | --- | --- | --- | --- | --- | --- |
| **Global/Regions/Countries** | **0-14 yrs** | | **15-49 yrs** | | **50-74 yrs** | | **≥75 yrs** | |
|  | **AAPC (95%CI, %)** | ***P*** | **AAPC (95%CI, %)** | ***P*** | **AAPC (95%CI, %)** | ***P*** | **AAPC (95%CI, %)** | ***P*** |
| Global | -0.70 (-0.73, -0.67) | ＜0.001 | -0.21 (-0.24, -0.17) | ＜0.001 | -0.24 (-0.29, -0.2) | ＜0.001 | 0.15 (0.12, 0.17) | ＜0.001 |
| **SDI Regions** |  |  |  |  |  |  |  |  |
| High SDI | -0.53 (-0.58, -0.47) | ＜0.001 | -0.13 (-0.17, -0.09) | ＜0.001 | -0.47 (-0.49, -0.45) | ＜0.001 | -0.17 (-0.20, -0.14) | ＜0.001 |
| High-middle SDI | -1.01 (-1.07, -0.94) | ＜0.001 | -0.13 (-0.17, -0.1) | ＜0.001 | -0.24 (-0.30, -0.18) | ＜0.001 | 0.58 (0.53, 0.63) | ＜0.001 |
| Middle SDI | -0.92 (-1.00, -0.84) | ＜0.001 | -0.05 (-0.08, -0.03) | ＜0.001 | -0.03 (-0.06, 0.01) | 0.154 | 0.59 (0.55, 0.63) | ＜0.001 |
| Low-middle SDI | -0.21 (-0.23, -0.19) | ＜0.001 | -0.03 (-0.05, -0.02) | ＜0.001 | -0.22 (-0.23, -0.21) | ＜0.001 | -0.08 (-0.09, -0.07) | ＜0.001 |
| Low SDI | -0.24 (-0.25, -0.22) | ＜0.001 | -0.42 (-0.44, -0.41) | ＜0.001 | -0.54 (-0.56, -0.52) | ＜0.001 | -0.27 (-0.29, -0.24) | ＜0.001 |
| **East Asia** |  |  |  |  |  |  |  |  |
| China | -1.22 (-1.27, -1.17) | ＜0.001 | 0.17 (0.08, 0.26) | ＜0.001 | 0.34 (0.29, 0.38) | ＜0.001 | 1.09 (1.05, 1.14) | ＜0.001 |
| **Central Asia** |  |  |  |  |  |  |  |  |
| Armenia | 0.10 (-0.02, 0.23) | 0.110 | 0.04 (-0.04, 0.12) | 0.338 | -0.32 (-0.48, -0.16) | ＜0.001 | -0.05 (-0.15, 0.06) | 0.384 |
| Azerbaijan | -0.01 (-0.09, 0.07) | 0.744 | 0.13 (0.06, 0.20) | ＜0.001 | -0.37 (-0.44, -0.29) | ＜0.001 | 0.88 (0.74, 1.01) | ＜0.001 |
| Georgia | -0.51 (-0.68, -0.34) | ＜0.001 | -0.26 (-0.3, -0.21) | ＜0.001 | 0.17 (0.13, 0.22) | ＜0.001 | 0.01 (-0.04, 0.06) | 0.697 |
| Kazakhstan | -0.85 (-1.00, -0.70) | ＜0.001 | -0.37 (-0.53, -0.2) | ＜0.001 | -0.66 (-0.76, -0.57) | ＜0.001 | -0.09 (-0.13, -0.05) | ＜0.001 |
| Kyrgyzstan | -0.50 (-0.57, -0.43) | ＜0.001 | -0.80 (-0.87, -0.73) | ＜0.001 | -1.28 (-1.38, -1.17) | ＜0.001 | -0.9 (-1.00, -0.80) | ＜0.001 |
| Mongolia | -1.31 (-1.49, -1.14) | ＜0.001 | 0.44 (0.39, 0.49) | ＜0.001 | -0.12 (-0.18, -0.05) | ＜0.001 | 0.79 (0.75, 0.83) | ＜0.001 |
| Tajikistan | -0.15 (-0.24, -0.07) | ＜0.001 | -0.07 (-0.15, 0.01) | 0.104 | -0.48 (-0.55, -0.41) | ＜0.001 | 0.39 (0.26, 0.53) | ＜0.001 |
| Turkmenistan | 0.09 (0.02, 0.17) | 0.019 | 1.23 (1.20, 1.27) | ＜0.001 | 0.51 (0.47, 0.55) | ＜0.001 | 0.57 (0.50, 0.64) | ＜0.001 |
| Uzbekistan | 0.06 (-0.01, 0.12) | 0.094 | 0.57 (0.47, 0.66) | ＜0.001 | -0.34 (-0.38, -0.29) | ＜0.001 | 0.72 (0.65, 0.79) | ＜0.001 |
| **South Asia** |  |  |  |  |  |  |  |  |
| Bangladesh | -0.10 (-0.14, -0.06) | ＜0.001 | 0.17 (0.13, 0.21) | ＜0.001 | -0.34 (-0.37, -0.32) | ＜0.001 | 0 (-0.05, 0.04) | 0.873 |
| Bhutan | -0.23 (-0.28, -0.17) | ＜0.001 | 0.20 (0.10, 0.29) | ＜0.001 | -0.40 (-0.43, -0.37) | ＜0.001 | 0.26 (0.22, 0.29) | ＜0.001 |
| India | -0.23 (-0.25, -0.21) | ＜0.001 | -0.06 (-0.09, -0.03) | ＜0.001 | -0.23 (-0.24, -0.21) | ＜0.001 | -0.02 (-0.06, 0.02) | 0.347 |
| Nepal | -0.27 (-0.28, -0.26) | ＜0.001 | -0.42 (-0.46, -0.37) | ＜0.001 | -0.44 (-0.47, -0.41) | ＜0.001 | -0.11 (-0.13, -0.09) | ＜0.001 |
| Pakistan | -0.29 (-0.31, -0.26) | ＜0.001 | 0.11 (0.08, 0.13) | ＜0.001 | -0.37 (-0.39, -0.35) | ＜0.001 | -0.13 (-0.16, -0.10) | ＜0.001 |
| **Southeast Asia** |  |  |  |  |  |  |  |  |
| Cambodia | -0.29 (-0.36, -0.21) | ＜0.001 | 0.03 (-0.01, 0.07) | 0.099 | -0.26 (-0.29, -0.22) | ＜0.001 | 0.12 (0.08, 0.15) | ＜0.001 |
| Indonesia | -1.11 (-1.14, -1.08) | ＜0.001 | -0.19 (-0.22, -0.16) | ＜0.001 | -0.23 (-0.27, -0.2) | ＜0.001 | 0.41 (0.39, 0.44) | ＜0.001 |
| Lao People's Democratic Republic | -0.33 (-0.36, -0.29) | ＜0.001 | -0.13 (-0.18, -0.09) | ＜0.001 | -0.58 (-0.62, -0.54) | ＜0.001 | -0.01 (-0.05, 0.02) | 0.411 |
| Malaysia | -0.11 (-0.14, -0.08) | ＜0.001 | -0.11 (-0.16, -0.07) | ＜0.001 | -0.25 (-0.30, -0.20) | ＜0.001 | -0.06 (-0.11, 0) | 0.056 |
| Maldives | -1.10 (-1.16, -1.04) | ＜0.001 | -0.88 (-0.97, -0.8) | ＜0.001 | -1.95 (-2.06, -1.84) | ＜0.001 | -1.00 (-1.04, -0.96) | ＜0.001 |
| Myanmar | -0.56 (-0.61, -0.51) | ＜0.001 | -0.07 (-0.11, -0.03) | 0.001 | -0.73 (-0.79, -0.66) | ＜0.001 | -0.08 (-0.11, -0.06) | ＜0.001 |
| Philippines | -0.30 (-0.32, -0.28) | ＜0.001 | 0.94 (0.91, 0.98) | ＜0.001 | 0.89 (0.81, 0.97) | ＜0.001 | 0.08 (0.05, 0.11) | ＜0.001 |
| Sri Lanka | -0.54 (-0.60, -0.48) | ＜0.001 | -0.25 (-0.29, -0.21) | ＜0.001 | -0.40 (-0.47, -0.34) | ＜0.001 | -0.30 (-0.34, -0.27) | ＜0.001 |
| Thailand | -0.63 (-0.69, -0.57) | ＜0.001 | 0.32 (0.25, 0.39) | ＜0.001 | -0.60 (-0.67, -0.52) | ＜0.001 | -0.36 (-0.41, -0.32) | ＜0.001 |
| Viet Nam | -0.11 (-0.20, -0.03) | 0.007 | 0.68 (0.64, 0.72) | ＜0.001 | 0 (-0.06, 0.06) | 0.919 | 0.06 (0.01, 0.11) | 0.012 |
| **High-income Asia pacific** |  |  |  |  |  |  |  |  |
| Brunei Darussalam | -0.38 (-0.41, -0.34) | ＜0.001 | -0.68 (-0.79, -0.58) | ＜0.001 | -1.76 (-1.90, -1.62) | ＜0.001 | -1.65 (-1.74, -1.57) | ＜0.001 |
| Singapore | -0.92 (-1.04, -0.79) | ＜0.001 | -0.44 (-0.50, -0.39) | ＜0.001 | -2.15 (-2.37, -1.93) | ＜0.001 | -2.11 (-2.19, -2.03) | ＜0.001 |
| **North Africa and Middle East** |  |  |  |  |  |  |  |  |
| Afghanistan | -0.94 (-1.08, -0.8) | ＜0.001 | -0.89 (-1.08, -0.69) | ＜0.001 | -0.80 (-0.85, -0.76) | ＜0.001 | -0.17 (-0.22, -0.13) | ＜0.001 |
| Bahrain | -0.66 (-0.74, -0.58) | ＜0.001 | -0.53 (-0.65, -0.4) | ＜0.001 | -1.03 (-1.15, -0.91) | ＜0.001 | -0.26 (-0.31, -0.21) | ＜0.001 |
| Egypt | -0.74 (-0.79, -0.69) | ＜0.001 | 0.08 (0.03, 0.14) | 0.003 | 0.40 (0.38, 0.42) | ＜0.001 | 0.79 (0.76, 0.82) | ＜0.001 |
| Iran (Islamic Republic of) | -1.30 (-1.38, -1.22) | ＜0.001 | -0.16 (-0.2, -0.12) | ＜0.001 | -0.61 (-0.67, -0.55) | ＜0.001 | 0.33 (0.31, 0.35) | ＜0.001 |
| Iraq | -0.28 (-0.33, -0.23) | ＜0.001 | -0.19 (-0.26, -0.12) | ＜0.001 | -0.42 (-0.45, -0.39) | ＜0.001 | 0.45 (0.40, 0.51) | ＜0.001 |
| Jordan | -0.17 (-0.26, -0.08) | ＜0.001 | -0.17 (-0.24, -0.1) | ＜0.001 | -0.48 (-0.57, -0.39) | ＜0.001 | 1.09 (0.99, 1.18) | ＜0.001 |
| Kuwait | -0.20 (-0.27, -0.13) | ＜0.001 | 0.29 (0.23, 0.35) | ＜0.001 | -0.40 (-0.59, -0.2) | ＜0.001 | 0.07 (0.01, 0.14) | 0.024 |
| Lebanon | -0.04 (-0.10, 0.03) | 0.249 | 0.14 (0.09, 0.2) | ＜0.001 | -0.07 (-0.10, -0.03) | ＜0.001 | 0.29 (0.25, 0.33) | ＜0.001 |
| Oman | -0.02 (-0.12, 0.08) | 0.657 | 0.31 (0.26, 0.36) | ＜0.001 | -0.64 (-0.67, -0.61) | ＜0.001 | 0.61 (0.55, 0.67) | ＜0.001 |
| Palestine | -0.37 (-0.43, -0.32) | ＜0.001 | -0.27 (-0.29, -0.24) | ＜0.001 | -0.75 (-0.80, -0.69) | ＜0.001 | -0.11 (-0.15, -0.08) | ＜0.001 |
| Qatar | -0.74 (-0.82, -0.67) | ＜0.001 | -0.68 (-0.78, -0.59) | ＜0.001 | -1.84 (-2.10, -1.58) | ＜0.001 | -1.29 (-1.52, -1.05) | ＜0.001 |
| Saudi Arabia | 0.27 (0.2, 0.33) | ＜0.001 | 0.50 (0.45, 0.54) | ＜0.001 | -0.54 (-0.64, -0.43) | ＜0.001 | 0.38 (0.28, 0.47) | ＜0.001 |
| Syrian Arab Republic | -1.03 (-1.16, -0.89) | ＜0.001 | -0.61 (-0.7, -0.52) | ＜0.001 | -0.93 (-0.99, -0.88) | ＜0.001 | -0.16 (-0.25, -0.08) | ＜0.001 |
| Türkiye | -1.19 (-1.30, -1.09) | ＜0.001 | -0.82 (-0.88, -0.75) | ＜0.001 | -1.27 (-1.34, -1.2) | ＜0.001 | -0.35 (-0.39, -0.31) | ＜0.001 |
| United Arab Emirates | -0.74 (-0.87, -0.6) | ＜0.001 | 0.22 (0.14, 0.3) | ＜0.001 | -0.87 (-0.94, -0.81) | ＜0.001 | -0.35 (-0.39, -0.3) | ＜0.001 |
| Yemen | -0.56 (-0.62, -0.49) | ＜0.001 | -0.32 (-0.35, -0.29) | ＜0.001 | -0.29 (-0.31, -0.27) | ＜0.001 | -0.03 (-0.07, 0.01) | 0.174 |
| **Central Europe** |  |  |  |  |  |  |  |  |
| Albania | -0.79 (-0.87, -0.71) | ＜0.001 | -0.19 (-0.26, -0.11) | ＜0.001 | -0.36 (-0.40, -0.32) | ＜0.001 | -0.23 (-0.26, -0.2) | ＜0.001 |
| Bosnia and Herzegovina | -0.40 (-0.50, -0.30) | ＜0.001 | 0.40 (0.34, 0.46) | ＜0.001 | 0.09 (-0.08, 0.27) | 0.295 | 0.21 (0.16, 0.26) | ＜0.001 |
| Bulgaria | -0.82 (-0.93, -0.71) | ＜0.001 | -0.04 (-0.08, 0) | 0.037 | -0.11 (-0.18, -0.03) | 0.005 | -0.51 (-0.54, -0.48) | ＜0.001 |
| Croatia | -0.92 (-0.97, -0.86) | ＜0.001 | -0.70 (-0.78, -0.61) | ＜0.001 | -0.68 (-0.76, -0.6) | ＜0.001 | 0.21 (0.14, 0.27) | ＜0.001 |
| Czechia | -0.69 (-0.75, -0.63) | ＜0.001 | -0.62 (-0.66, -0.57) | ＜0.001 | -1.00 (-1.08, -0.92) | ＜0.001 | -0.34 (-0.43, -0.26) | ＜0.001 |
| Hungary | -0.76 (-0.81, -0.72) | ＜0.001 | -1.11 (-1.20, -1.02) | ＜0.001 | -1.63 (-1.74, -1.53) | ＜0.001 | -1.09 (-1.20, -0.98) | ＜0.001 |
| Montenegro | -0.34 (-0.39, -0.29) | ＜0.001 | -0.13 (-0.21, -0.06) | 0.001 | -0.11 (-0.18, -0.05) | ＜0.001 | 0 (-0.08, 0.07) | 0.924 |
| North Macedonia | -0.73 (-0.82, -0.64) | ＜0.001 | -0.40 (-0.50, -0.29) | ＜0.001 | -0.55 (-0.63, -0.46) | ＜0.001 | -0.43 (-0.46, -0.4) | ＜0.001 |
| Poland | -0.87 (-0.92, -0.82) | ＜0.001 | -0.67 (-0.71, -0.62) | ＜0.001 | -0.47 (-0.54, -0.39) | ＜0.001 | 0.64 (0.57, 0.72) | ＜0.001 |
| Romania | -0.49 (-0.54, -0.43) | ＜0.001 | -0.21 (-0.34, -0.08) | 0.002 | -0.40 (-0.56, -0.25) | ＜0.001 | -0.11 (-0.13, -0.08) | ＜0.001 |
| Serbia | -0.86 (-0.98, -0.73) | ＜0.001 | -0.82 (-0.91, -0.72) | ＜0.001 | -0.99 (-1.06, -0.91) | ＜0.001 | -0.29 (-0.36, -0.21) | ＜0.001 |
| Slovakia | -0.46 (-0.55, -0.38) | ＜0.001 | -0.12 (-0.19, -0.06) | ＜0.001 | -0.86 (-1, -0.73) | ＜0.001 | -0.80 (-1.01, -0.59) | ＜0.001 |
| Slovenia | -1.15 (-1.24, -1.06) | ＜0.001 | -0.73 (-0.81, -0.66) | ＜0.001 | -1.14 (-1.22, -1.06) | ＜0.001 | -0.45 (-0.53, -0.36) | ＜0.001 |
| **Eastern Europe** |  |  |  |  |  |  |  |  |
| Belarus | -0.06 (-0.22, 0.1) | 0.442 | 0.24 (0.15, 0.32) | ＜0.001 | -0.25 (-0.37, -0.12) | ＜0.001 | 0.08 (-0.05, 0.21) | 0.244 |
| Estonia | -0.02 (-0.13, 0.09) | 0.715 | -0.62 (-0.69, -0.56) | ＜0.001 | -1.17 (-1.40, -0.95) | ＜0.001 | -0.16 (-0.32, 0) | 0.055 |
| Latvia | -0.18 (-0.24, -0.11) | ＜0.001 | -0.50 (-0.61, -0.4) | ＜0.001 | -0.26 (-0.36, -0.16) | ＜0.001 | 0.89 (0.81, 0.96) | ＜0.001 |
| Lithuania | -0.27 (-0.35, -0.19) | ＜0.001 | -0.14 (-0.22, -0.05) | 0.001 | -0.08 (-0.56, 0.41) | 0.761 | 0.86 (0.25, 1.48) | 0.006 |
| Republic of Moldova | 0.11 (0, 0.22) | 0.047 | 0.11 (0.05, 0.17) | 0.001 | -0.20 (-0.26, -0.13) | ＜0.001 | -0.06 (-0.17, 0.06) | 0.352 |
| Russian Federation | -0.39 (-0.48, -0.3) | ＜0.001 | 0.14 (0.05, 0.23) | 0.003 | -0.19 (-0.29, -0.10) | ＜0.001 | 0.13 (-0.07, 0.32) | 0.213 |
| Ukraine | 0 (-0.23, 0.24) | 0.968 | 0.39 (0.32, 0.46) | ＜0.001 | -0.40 (-0.46, -0.34) | ＜0.001 | -0.63 (-0.79, -0.47) | ＜0.001 |
| **Western Europe** |  |  |  |  |  |  |  |  |
| Cyprus | -0.76 (-0.82, -0.7) | ＜0.001 | -0.40 (-0.46, -0.34) | ＜0.001 | -1.75 (-1.84, -1.65) | ＜0.001 | -2.34 (-2.52, -2.16) | ＜0.001 |
| Greece | -0.50 (-0.56, -0.45) | ＜0.001 | -0.30 (-0.34, -0.26) | ＜0.001 | -0.79 (-0.85, -0.72) | ＜0.001 | -0.64 (-0.67, -0.60) | ＜0.001 |
| Israel | -0.76 (-0.80, -0.71) | ＜0.001 | -0.28 (-0.35, -0.20) | ＜0.001 | -1.12 (-1.19, -1.04) | ＜0.001 | -1.05 (-1.1, -1.00) | ＜0.001 |

AAPC: average annual percentage change; CI: confidence interval

| Table S5 The long-term trends of stroke-related YLDs rate attributable to risk factors from 1990 to 2021 for “the Belt & Road” countries | | | | | | |
| --- | --- | --- | --- | --- | --- | --- |
|  | **Metabolic risks** | | **Environmental/occupational risks** | | **Behavioral risks** | |
| **Global/Regions/Countries** | **AAPC (95%CI, %)** | ***P*** | **AAPC (95%CI, %)** | ***P*** | **AAPC (95%CI, %)** | ***P*** |
| Global | -0.12(-0.13, -0.11) | ＜0.001 | -0.92 (-1.01, -0.83) | ＜0.001 | -0.53 (-0.55, -0.51) | ＜0.001 |
| **SDI Regions** |  |  |  |  |  |  |
| High SDI | -0.61(-0.67, -0.55) | ＜0.001 | -1.54(-1.60, -1.48) | ＜0.001 | -0.81(-0.83, -0.78) | ＜0.001 |
| High-middle SDI | -0.03(-0.05, -0.01) | 0.001 | -1.19(-1.43, -0.94) | ＜0.001 | -0.40(-0.42, -0.37) | ＜0.001 |
| Middle SDI | 0.31(0.28, 0.34) | ＜0.001 | -1.13(-1.22, -1.05) | ＜0.001 | -0.36(-0.38, -0.33) | ＜0.001 |
| Low-middle SDI | 0.01(-0.00, 0.02) | 0.097 | -0.79(-0.87, -0.72) | ＜0.001 | -0.60(-0.61, -0.58) | ＜0.001 |
| Low SDI | -0.21(-0.23, -0.19) | ＜0.001 | -0.53(-0.59, -0.48) | ＜0.001 | -0.67(-0.69, -0.65) | ＜0.001 |
| **East Asia** |  |  |  |  |  |  |
| China | 0.83(0.79, 0.88) | ＜0.001 | -0.89(-0.97, -0.82) | ＜0.001 | 0.09(0.04, 0.13) | ＜0.001 |
| **Central Asia** |  |  |  |  |  |  |
| Armenia | -0.50(-0.55, -0.46) | ＜0.001 | -0.89(-1.11, -0.68) | ＜0.001 | -0.74(-0.77, -0.72) | ＜0.001 |
| Azerbaijan | -0.04(-0.08, 0.00) | 0.056 | -1.32(-1.49, -1.15) | ＜0.001 | -0.46(-0.48, -0.43) | ＜0.001 |
| Georgia | -0.21(-0.22, -0.19) | ＜0.001 | -0.64(-0.77, -0.51) | ＜0.001 | 0.02(-0.02, 0.05) | 0.359 |
| Kazakhstan | -0.60(-0.66, -0.55) | ＜0.001 | -1.27(-1.59, -0.95) | ＜0.001 | -1.30(-1.37, -1.23) | ＜0.001 |
| Kyrgyzstan | -1.18(-1.25, -1.10) | ＜0.001 | -1.66(-1.73, -1.58) | ＜0.001 | -1.31(-1.36, -1.26) | ＜0.001 |
| Mongolia | 0.27(0.21, 0.32) | ＜0.001 | -1.18(-1.25, -1.12) | ＜0.001 | -0.20(-0.26, -0.15) | ＜0.001 |
| Tajikistan | -0.15 (-0.20, -0.09) | ＜0.001 | -0.81(-0.98, -0.65) | ＜0.001 | -1.00(-1.06, -0.94) | ＜0.001 |
| Turkmenistan | 0.73(0.69, 0.77) | ＜0.001 | 0.62(0.34, 0.90) | ＜0.001 | 0.36(0.33, 0.40) | ＜0.001 |
| Uzbekistan | 0.18(0.16, 0.20) | ＜0.001 | -0.64(-0.84, -0.45) | ＜0.001 | -0.21(-0.24, -0.17) | ＜0.001 |
| **South Asia** |  |  |  |  |  |  |
| Bangladesh | 0.12(0.02, 0.23) | 0.019 | -0.57(-0.67, -0.46) | ＜0.001 | -0.73(-0.76, -0.69) | ＜0.001 |
| Bhutan | -0.16(-0.20, -0.12) | ＜0.001 | -1.94(-2.38, -1.50) | ＜0.001 | -0.92(-0.96, -0.88) | ＜0.001 |
| India | -0.11(-0.12, -0.09) | ＜0.001 | -0.82(-1.00, -0.65) | ＜0.001 | -0.55(-0.56, -0.54) | ＜0.001 |
| Nepal | -0.51(-0.55, -0.46) | ＜0.001 | -0.66(-0.77, -0.56) | ＜0.001 | -1.24(-1.28, -1.20) | ＜0.001 |
| Pakistan | 0.07(0.06, 0.08) | ＜0.001 | -0.64(-0.77, -0.51) | ＜0.001 | -0.55(-0.57, -0.53) | ＜0.001 |
| **Southeast Asia** |  |  |  |  |  |  |
| Cambodia | -0.21(-0.23, -0.18) | ＜0.001 | -0.49(-0.52, -0.46) | ＜0.001 | -0.33(-0.36, -0.29) | ＜0.001 |
| Indonesia | -0.00(-0.02, 0.01) | 0.472 | -2.09(-2.23, -1.95) | ＜0.001 | -0.70(-0.73, -0.66) | ＜0.001 |
| Lao People's Democratic Republic | -0.25(-0.28, -0.23) | ＜0.001 | -0.86(-0.90, -0.82) | ＜0.001 | -1.06(-1.09, -1.03) | ＜0.001 |
| Malaysia | -0.04(-0.06, -0.01) | 0.008 | -1.76(-2.10, -1.42) | ＜0.001 | -0.98(-1.01, -0.94) | ＜0.001 |
| Maldives | -1.50(-1.56, -1.44) | ＜0.001 | -5.21(-5.30, -5.12) | ＜0.001 | -2.10(-2.22, -1.99) | ＜0.001 |
| Myanmar | -0.34(-0.37, -0.32) | ＜0.001 | -0.95(-0.97, -0.93) | ＜0.001 | -1.69(-1.74, -1.65) | ＜0.001 |
| Philippines | 0.64(0.62, 0.66) | ＜0.001 | -0.48(-0.58, -0.37) | ＜0.001 | 0.05(0.03, 0.08) | ＜0.001 |
| Sri Lanka | -0.23(-0.26, -0.21) | ＜0.001 | -2.43(-2.49, -2.37) | ＜0.001 | -1.32(-1.38, -1.26) | ＜0.001 |
| Thailand | -0.43(-0.47, -0.40) | ＜0.001 | -2.32(-2.39, -2.24) | ＜0.001 | -1.34(-1.37, -1.31) | ＜0.001 |
| Viet Nam | 0.40(0.36, 0.43) | ＜0.001 | -1.54(-1.62, -1.47) | ＜0.001 | -0.30(-0.33, -0.27) | ＜0.001 |
| **High-income Asia pacific** |  |  |  |  |  |  |
| Brunei Darussalam | -1.73(-1.78, -1.68) | ＜0.001 | -2.26(-2.46, -2.07) | ＜0.001 | -2.16(-2.19, -2.12) | ＜0.001 |
| Singapore | -2.62(-2.71, -2.52) | ＜0.001 | -3.84(-4.58, -3.09) | ＜0.001 | -2.67(-2.77, -2.57) | ＜0.001 |
| **North Africa and Middle East** | |  |  |  |  |  |
| Afghanistan | -0.35(-0.36, -0.33) | ＜0.001 | -0.73(-0.82, -0.64) | ＜0.001 | -0.23(-0.28, -0.17) | ＜0.001 |
| Bahrain | -0.65(-0.68, -0.62) | ＜0.001 | -1.04(-1.10, -0.97) | ＜0.001 | -0.95(-0.99, -0.91) | ＜0.001 |
| Egypt | 0.84(0.79, 0.89) | ＜0.001 | 0.20(-0.10, 0.50) | 0.19 | 0.18(0.16, 0.20) | ＜0.001 |
| Iran (Islamic Republic of) | -0.28(-0.31, -0.26) | ＜0.001 | -0.69(-0.79, -0.60) | ＜0.001 | -0.58(-0.61, -0.56) | ＜0.001 |
| Iraq | -0.08(-0.11, -0.05) | ＜0.001 | -0.69(-0.78, -0.61) | ＜0.001 | -0.57(-0.60, -0.55) | ＜0.001 |
| Jordan | 0.05(-0.01, 0.10) | 0.105 | 0.38(0.18, 0.57) | ＜0.001 | -0.41(-0.46, -0.36) | ＜0.001 |
| Kuwait | -0.17(-0.28, -0.05) | 0.005 | -0.38(-0.63, -0.12) | 0.003 | -0.39(-0.45, -0.33) | ＜0.001 |
| Lebanon | 0.22(0.18, 0.26) | ＜0.001 | 0.06(-0.16, 0.29) | 0.581 | 0.16(0.13, 0.19) | ＜0.001 |
| Oman | 0.42(0.37, 0.48) | ＜0.001 | -0.55(-0.97, -0.13) | 0.011 | -0.52(-0.56, -0.48) | ＜0.001 |
| Palestine | -0.39(-0.43, -0.35) | ＜0.001 | -0.82(-1.09, -0.55) | ＜0.001 | -0.99(-1.03, -0.95) | ＜0.001 |
| Qatar | -1.39(-1.59, -1.19) | ＜0.001 | -1.66(-1.76, -1.56) | ＜0.001 | -1.56(-1.77, -1.35) | ＜0.001 |
| Saudi Arabia | 0.03(-0.03, 0.08) | 0.301 | 0.06(-0.09, 0.21) | 0.429 | 0.12(0.08, 0.16) | ＜0.001 |
| Syrian Arab Republic | -0.68(-0.71, -0.65) | ＜0.001 | -1.26(-1.50, -1.02) | ＜0.001 | -1.13(-1.20, -1.07) | ＜0.001 |
| Türkiye | -1.07(-1.14, -0.99) | ＜0.001 | -1.64(-2.10, -1.18) | ＜0.001 | -1.71(-1.79, -1.64) | ＜0.001 |
| United Arab Emirates | -0.64(-0.67, -0.61) | ＜0.001 | -1.00(-1.39, -0.61) | ＜0.001 | -0.49(-0.52, -0.47) | ＜0.001 |
| Yemen | -0.05(-0.07, -0.02) | ＜0.001 | -0.97 (-1.12, -0.83) | ＜0.001 | -0.29(-0.31, -0.27) | ＜0.001 |
| **Central Europe** |  |  |  |  |  |  |
| Albania | -0.37(-0.44, -0.30) | ＜0.001 | -3.08(-3.17, -2.99) | ＜0.001 | -0.58(-0.63, -0.52) | ＜0.001 |
| Bosnia and Herzegovina | -0.14(-0.17, -0.12) | ＜0.001 | -1.87(-1.93, -1.81) | ＜0.001 | -0.37(-0.40, -0.34) | ＜0.001 |
| Bulgaria | -0.32(-0.34, -0.31) | ＜0.001 | -1.77(-1.86, -1.67) | ＜0.001 | -0.62(-0.66, -0.59) | ＜0.001 |
| Croatia | -0.68(-0.71, -0.66) | ＜0.001 | -2.06(-2.23, -1.90) | ＜0.001 | -1.06(-1.11, -1.02) | ＜0.001 |
| Czechia | -0.92(-0.98, -0.86) | ＜0.001 | -2.65(-2.78, -2.52) | ＜0.001 | -1.26(-1.29, -1.24) | ＜0.001 |
| Hungary | -1.64(-1.73, -1.55) | ＜0.001 | -3.35(-3.46, -3.24) | ＜0.001 | -2.04(-2.13, -1.94) | ＜0.001 |
| Montenegro | -0.32(-0.41, -0.24) | ＜0.001 | -1.87(-2.01, -1.74) | ＜0.001 | -0.61(-0.70, -0.52) | ＜0.001 |
| North Macedonia | -0.67(-0.68, -0.66) | ＜0.001 | -2.15(-2.19, -2.10) | ＜0.001 | -0.98(-1.01, -0.94) | ＜0.001 |
| Poland | -0.56(-0.60, -0.53) | ＜0.001 | -2.42(-2.57, -2.27) | ＜0.001 | -0.98(-1.00, -0.95) | ＜0.001 |
| Romania | -0.57(-0.62, -0.53) | ＜0.001 | -2.62(-2.70, -2.54) | ＜0.001 | -0.80(-0.85, -0.74) | ＜0.001 |
| Serbia | -0.96(-1.01, -0.91) | ＜0.001 | -2.39(-2.48, -2.31) | ＜0.001 | -1.20(-1.24, -1.17) | ＜0.001 |
| Slovakia | -0.99(-1.07, -0.90) | ＜0.001 | -2.40(-2.57, -2.22) | ＜0.001 | -1.18(-1.36, -1.00) | ＜0.001 |
| Slovenia | -1.15(-1.21, -1.09) | ＜0.001 | -2.91(-3.24, -2.58) | ＜0.001 | -1.51(-1.59, -1.43) | ＜0.001 |
| **Eastern Europe** |  |  |  |  |  |  |
| Belarus | -0.28(-0.34, -0.21) | ＜0.001 | -2.37(-2.62, -2.11) | ＜0.001 | -0.51(-0.61, -0.40) | ＜0.001 |
| Estonia | -1.15(-1.19, -1.12) | ＜0.001 | -4.39(-5.13, -3.65) | ＜0.001 | -1.36(-1.45, -1.28) | ＜0.001 |
| Latvia | -0.28(-0.36, -0.19) | ＜0.001 | -2.98(-3.29, -2.68) | ＜0.001 | -0.48(-0.60, -0.37) | ＜0.001 |
| Lithuania | -0.07(-0.35, 0.21) | 0.627 | -2.82(-3.07, -2.56) | ＜0.001 | -0.25(-0.53, 0.04) | 0.09 |
| Republic of Moldova | -0.12(-0.16, -0.09) | ＜0.001 | -2.99(-3.24, -2.74) | ＜0.001 | -0.21(-0.25, -0.16) | ＜0.001 |
| Russian Federation | -0.34(-0.38, -0.31) | ＜0.001 | -2.68(-3.02, -2.35) | ＜0.001 | -0.56(-0.60, -0.53) | ＜0.001 |
| Ukraine | -0.49(-0.54, -0.44) | ＜0.001 | -2.57(-2.71, -2.43) | ＜0.001 | -0.77(-0.86, -0.68) | ＜0.001 |
| **Western Europe** |  |  |  |  |  |  |
| Cyprus | -1.76(-1.81, -1.71) | ＜0.001 | -2.85(-3.04, -2.66) | ＜0.001 | -1.60(-1.68, -1.52) | ＜0.001 |
| Greece | -1.06(-1.12, -1.00) | ＜0.001 | -2.13(-2.18, -2.08) | ＜0.001 | -1.13(-1.16, -1.09) | ＜0.001 |
| Israel | -1.10(-1.20, -1.01) | ＜0.001 | -1.82(-1.97, -1.66) | ＜0.001 | -1.40(-1.52, -1.28) | ＜0.001 |

AAPC: average annual percentage change; CI: confidence interval
